# Switching off CK2-mediated activation of survivin offers new therapeutic opportunities in neuroblastoma

**DOI:** 10.1038/s12276-025-01628-5

**Published:** 2026-01-22

**Authors:** Giulia Cazzanelli, Andrea Dalle Vedove, Francesca Broso, Matteo Burigotto, Jacopo Zasso, Giuseppe Aiello, Francesca Zonta, Andrea Astolfi, Maria Letizia Barreca, Maria Ruzzene, Luca Tiberi, Luca L. Fava, Alessandro Quattrone, Graziano Lolli

**Affiliations:** 1 Department of Cellular, Computational and Integrative Biology, University of Trento, Trento, Italy; 2 Department of Biomedical Sciences and CNR Institute of Neuroscience, University of Padua, Padua, Italy; 3 Department of Pharmaceutical Sciences, University of Perugia, Perugia, Italy

**Keywords:** Paediatric cancer, Drug development, Mitosis

## Abstract

CK2 is an antiapoptotic kinase overactive in various malignancies. Here we show that CK2 inhibition dramatically affects neuroblastoma growth both in vitro and in vivo. In particular, here we report on the identification of CK2-TN03, a CK2 inhibitor showing greater selectivity and cellular efficacy than silmitasertib, the only available clinical grade CK2 inhibitor with orphan status for cholangiocarcinoma and in clinical trials for medulloblastoma. CK2-TN03 acts by suppressing survivin, which is overexpressed in all high-risk neuroblastomas. Survivin function is affected by direct inhibition of its phosphorylation by CK2; its messenger RNA and protein levels are reduced through CK2 regulation of the MDM2/p53 balance via AKT1 and BRD4/MYCN. Accordingly, neuroblastoma cells persistently stall in mitosis before going to apoptosis. Finally, CK2-TN03 does not affect noncycling cells and significantly reduces tumor growth in mice xenografts without any apparent toxicity.

## New CK2 inhibitor shows promise against neuroblastoma tumors

Researchers have been exploring ways to inhibit CK2 to treat cancers such as neuroblastoma, a type of cancer that affects nerve cells. The study addresses the challenge of finding effective CK2 inhibitors. Researchers identified a new CK2 inhibitor called CK2-TN03. They tested its effects on various cancer cell lines, including neuroblastoma cells, using laboratory experiments. The study involved testing the compound’s ability to reduce cancer cell growth and induce cell death. They also examined how CK2-TN03 affects proteins involved in cell division and survival. Results showed CK2-TN03 effectively reduced tumor growth in neuroblastoma cells and was more potent than an existing inhibitor, CX-4945. It worked by disrupting the function of a protein called survivin, which helps cancer cells survive and divide. Researchers concluded that CK2-TN03 has potential as a treatment for neuroblastoma and possibly other cancers.

This summary was initially drafted using artificial intelligence, then revised and fact-checked by the author.

## Introduction

The Ser/Thr kinase CK2 is an antiapoptotic cancer-sustaining protein^1^ kept inactive by an unusual self-inhibitory mechanism^2–4^. It is overexpressed/overactive in various cancers^1,5^, also promoting drug resistance^6^. The CK2 inhibitor CX-4945 (silmitasertib) was granted the Orphan Drug designation for cholangiocarcinoma in the USA and is in clinical trials for the treatment of various tumors^7–10^. The last added among these is medulloblastoma (MB), following the observed efficacy of CX-4945 in MB cell lines, where it caused a reduction in cell viability comparable to CK2 silencing^11,12^. In mice allografts, CX-4945 showed reduced tumor growth and enhanced survival. However, mice treatment was conducted at the high daily dosage of 75 mg/kg (37.5 mg/kg twice per day) in both studies, eventually reflecting a suboptimal pharmacokinetic profile for CX-4945^13^. Recently, the opportunity of targeting CK2 in cancer has been challenged by the observation that a specific CK2 inhibitor does not affect cancer growth when tested on a large cellular panel^14^. The CX-4945 efficacy was ascribed to multiple kinases inhibition (DYRKs and CLKs other than DAPK3, PIM1, HIPK3 and TBK1), which could suggest that a high dosage is needed to achieve such cross-reactivity. Almost concurrently, a promiscuous CK2/DYRK1/TNIK inhibitor was shown to inhibit growth of TNBC both in vitro and in vivo^15^. However, it is worth noting that CK2 knockout or silencing has been demonstrated in numerous reports (reviewed in ref. ^16^) to reduce growth and viability in various cell lines, making the argument controversial.

We identified a CK2 inhibitor (CK2-TN03), which, although less potent in kinase assay with respect to CX-4945, showed superior efficacy in cancerous cells. Most importantly, CK2-TN03 and CX-4945 induced cell death in neuroblastoma (NB) cells more dramatically than in MB cells. Very little evidence of CK2 functions in NB cell biology and malignancy has been reported^17–21^, and a clear-cut role of CK2 in NB etiology is here provided for the first time. CK2-TN03 caused long-term permanence of tetraploid NB cells in mitosis, which finally undergo apoptosis. Its mechanism of action relies on the inhibition of the CK2-mediated activation of survivin, which is overexpressed in all high-risk NB. Finally, CK2-TN03 induced a significant reduction of tumor growth in mice xenografts without any toxicity, thus encouraging new efforts toward evaluating the preclinical and clinical efficacy of CK2 inhibitors in NB.

## Materials and methods

### Compounds selection

The target section of the ChEMBL database was interrogated using the UniProt ID for CK2α (P68400) and retrieving the ID CHEMBL3629. Inhibitors with reported *K*_i_ (inhibition constant) values were ordered by decreasing potency, and compounds bearing a guaiacol headgroup and reported *K*_i_ values ≤20 nM were visually filtered and obtained through ChemSpace.

### Protein purification, X-ray crystallography and activity assay

CK2 alpha was produced as previously reported^22^. Briefly, after expression in *E. coli* BL21-DE3, human CK2 alpha (amino acids 1–336) was purified by sequential affinity, anion exchange and size-exclusion chromatography (HiTrap Heparin, MonoQ and Superdex 75). The protein was concentrated to 10 mg/ml and frozen in liquid nitrogen.

Crystals of apo CK2 and soaking of inhibitors were obtained as previously described^23^. Diffraction data were collected at the Elettra Synchrotron Light Source (Trieste, Italy), XRD1 beamline. Data were processed and structures were solved as described elsewhere^23^. Data collection and refinement statistics are reported in Supplementary Tables 1–3. Electron densities (*F*_o_ − *F*_c_ polder OMIT map) for the bound inhibitors are shown in Supplementary Fig. 1.

The half-maximum inhibitory concentration (IC_50_) values (concentrations inducing 50% inhibition) of the inhibitors were determined by CK2 activity assays in the presence of [γ-^32^P]ATP, the model peptide R_3_AD_2_SD_5_ (CK2-tide) as substrate, and increasing concentrations of the inhibitors, as described in ref. ^24^.

The kinase inhibition profile was determined at Reaction Biology Europe GmbH by measuring residual activity values at 5× and 10× the IC_50_ value (Table 1) in 345 wild-type protein kinase assays. The final DMSO concentration in all reaction cocktails was 1%. A radiometric protein kinase assay (^33^PanQinase Activity Assay) was used for measuring the kinase activity. The assay for all protein kinases contained 70 mM HEPES–NaOH pH 7.5, 3 mM MgCl_2_, 3 mM MnCl_2_, 3 μM Na-orthovanadate, 1.2 mM DTT, 50 μg/ml PEG_20000_, ATP (variable amounts, corresponding to the apparent ATP-K_m_ of the respective kinase), [γ-^33^P]-ATP (approximately 8 × 10^5^ cpm per well), protein kinase and substrate. The protein kinase reaction cocktails were incubated at 30 °C for 60 min. The reaction was stopped with 50 μl of 2% (v/v) H_3_PO_4_, and the plates were aspirated and washed two times with 200 μl 0.9% (w/v) NaCl. Incorporation of ^33^P_i_ (counting of ‘cpm’) was determined with a microplate scintillation counter (Microbeta, Wallac). All protein kinase assays were performed with a BeckmanCoulter Biomek 2000/SL robotic system. For each kinase, the median value of the cpm of three wells was defined as ‘low control’ (*n* = 3). This value reflects unspecific binding of radioactivity to the plate in the absence of a protein kinase but in the presence of the substrate. In addition, for each kinase the median value of the cpm of three other wells was taken as the ‘high control’, that is, full activity in the absence of any inhibitor (*n* = 3). The difference between high and low control of each enzyme was taken as 100% activity.

**Table 1:** Structures, properties and activities for the selected CK2 inhibitors.

	Structure	IC_50_ (nM)	Ki (nM)	pKa^d^	Percent charged at pH 7.4^d^
CK2-TN01		89 (±36)	5^a^	7.9	25%
CK2-TN02		62 (±4)	4^a^	6.2	94%
CK2-TN03		165 (±39)	20^a^	9.7	0%
CX-4945		1^b^; 14.7^c^	1^a^; 0.38^b^	3.0	100%

^a^In the ChEMBL database (https://www.ebi.ac.uk/chembl/); ^b^in ref. ^68^; ^c^in ref. ^69^; ^d^calculated at chemicalize.com (ChemAxon).

As part of the data evaluation the low control of each kinase was subtracted from the high control value as well as from their corresponding ‘compound values’. The residual activity (in percentage) for each compound well was calculated by using the following formula: residual activity (%) = 100 × ((signal of compound – low control)/(high control – low control)).

The selectivity score, according to Karaman et al.^25^, is a compound concentration-dependent parameter describing the portion of kinases, which are inhibited to more than a predefined degree (for example, more than 50%), in relation to all tested kinases of the particular project. The selectivity score of the compound at the tested concentrations was calculated for a residual activity <50%, that is, an inhibition of >50%. The selectivity was calculated by using the following formula: selectivity score = (count of data points <50%)/(total number of data points).

### Molecular Docking

The X-ray structure of CK2-TN03 complexed with CK2 was prepared using Schrödinger’s Protein Preparation Wizard^26,27^ to obtain satisfactory starting structures for modeling studies. The complex was preprocessed as follows: (1) hydrogen atoms were added and bond orders were assigned, (2) the missing side chains and the missed residues were filled, (3) the protein was capped with acetyl and *N*-methylamine groups.

The cocrystallized ligand was used as template to generate the three CK2-TN03 tautomers T1–3. For each tautomer, a complex with no water molecule and a complex with the conserved water molecule W1 were created, as the presence of structural water molecules can substantially impact docking results^28–31^. In total, six different complexes were collected. The H-bond network was optimized using PROPKA^32^ for the assignment of the residue protonation states (pH 7.0), and finally, the complexes were submitted to a restrained minimization (OPLS4 force field^33^), which was stopped when RMSD of heavy atoms reached 0.30 Å.

CK2-TN03 analogs were built by using the ‘R-Group creator’ tool in Maestro^34^. Once generated the compounds were prepared with LigPrep keeping constant the tautomeric/protomeric state of the CK2-TN03 thiazolidinone core. The ligands were minimized using the OPLS4 force field.

The grids were generated by using the ‘Receptor Grid Generation’ tool of Maestro^34^. The position of the cocrystallized ligand was used as reference to center the grid of each complex. The docking space was defined as a cubic box (28 Å outerbox), with an inner cubic box (14 Å) defining the region where the centroid of the ligand had to be located. The docking experiments were performed using the Glide program^35,36^ using the standard precision protocol and applying the default settings. One pose for each ligand was created.

### Cell culture

The MB cell line DAOY and the glioblastoma cell line U87 were cultured in DMEM medium. The NB cell line CHP-212 was cultured in MEM/F12 (1:1) medium supplemented with 1× non essential amino acids; SK-N-AS and SH-SY5Y cells were cultured in DMEM medium; CHP-134 and SK-N-FI were cultured in RPMI 1640 medium; IMR-32 cells were cultured in MEM medium. All mediums contained 10% FBS, 1% penicillin–streptomycin, 2 mM L-glutamine. All cell lines were regularly tested for mycoplasma contamination.

For the differentiation of SH-SY5Y cell line, cells were allowed to attach overnight and then the medium was changed, reducing the FBS to 1% and adding 10 μM retinoic acid (RA). After 72 h, the medium was refreshed. A total of 72 h later, RA was excluded from the medium, and 50 ng/ml of BDNF were added instead. The differentiated cells were analyzed (morphology, viability and protein extraction) 48 h after the addition of BDNF.

### Cell viability and caspase 3/7 activity

Cells were counted with a hemocytometer and seeded in triplicates in 96-well plates (1 × 10^4^ cells per well) and let adhere overnight at 37 °C, 5% CO_2_. The following day, the medium was replaced adding either the carrier (0.05% DMSO) or the inhibitor (0.2, 0.5, 1.0, 2.5, 5.0, 10.0, 25.0 μM CK2-TN03 and 0.5, 1.0, 2.5, 5.0, 10.0, 17.5, 25.0 μM CX-4945 for viability; 0.35, 0.5, 1.0 μM CK2-TN03 for caspases 3/7 activity) resuspended in the same amount of carrier. In the case of double treatment (CK2-TN03 and Q-VD-OPh), the two molecules were added at the same time and the amount of carrier in the untreated sample was adjusted to be the same as in the treated one.

Cell viability was measured after 48 h by adding 10% resazurin sodium salt solution (0.03 mg/ml powder from Sigma-Aldrich dissolved in PBS) directly into each well and incubating the cells for 4 h at 37 °C, 5% CO_2_. The fluorescence at 570/600 nm was measured using Varioskan LUX (Thermo Scientific) plate reader. The viability was calculated as percentage compared to the sample treated with the carrier only, considered 100% viable. The log(inhibitor) versus response, variable slope (four parameters) model of the Graphpad Prism software was used to determine the half-maximum effective concentration (EC_50_) of each replicate, which were then averaged.

Caspases 3/7 activity was measured by adding the suggested amount of CellEvent Caspase 3/7 Green ReadyProbes Reagent (Invitrogen) to the medium containing the inhibitor/carrier. Cells were incubated for 15 min at 37 °C, 5% CO_2_, and then placed in the Incucyte S3 (Sartorius). The activation of caspases was monitored by taking a picture every 2 h for 48 h totally (10× magnification; five fields per well) in brightfield and in green fluorescence (excitation/emission: 502/530). The Incucyte S3 software was then used to count the green events, representative of caspases 3/7 activation.

### Cell cycle

A total of 1,200,000 cells per condition were seeded in six-well plates (300,000 cells per well, four wells per condition pulled together at the end of the experiment) and let attach overnight at 37 °C, 5% CO_2_. The following day the medium was replaced with medium containing either the carrier (0.05% DMSO) or the inhibitor (0.5–1.0 μM CK2-TN03) resuspended in the same amount of carrier. The cells were collected from both the plate surface by trypsin and the supernatant medium and washed in cold PBS. Cells were fixed in cold 70% ethanol, adding it drop by drop while vortexing, and then, they were incubated at 4 °C for at least 24 h. The cell pellet was washed twice in PBS and resuspended in FxCycle PI/RNase Staining Solution (Invitrogen). The samples were incubated in the dark for 15 min and analyzed using BD FACSCantoA flow cytometer at 535/617 nm excitation/emission. A total of 10,000–20,000 cells were examined per sample. The data were analyzed using FlowJo and ModFit LT software (Supplementary Fig. 2).

### Live imaging

Teh cells were counted with a hemocytometer and seeded in an Ibidi chambered coverslip with eight wells (4 × 10^4^ cells per well) and let adhere for 48 h at 37 °C, 5% CO_2_. The medium was changed with medium containing the appropriate inhibitor (0.5 μM CK2-TN03; 0.2 μg/ml reversine; combination of the two) and 1 μM SiR-DNA staining. The cells were incubated for further 4 h before starting live imaging. Cells were imaged using a Nikon Eclipse Ti2+ spinning disc. An image (three fields per well) was taken every 5 min for 24 h totally at 20× magnification in brightfield and far red fluorescence. The analysis was performed manually on 200–300 cells per field.

### Western blot

Total protein content was extracted from cells treated for 48 h with 0.5 μM CK2-TN03 or with the carrier. Cells were previously counted and plated at a fixed amount (1,720,000 cells per 100 mm plate). Cells were lysed using RIPA buffer and protein concentration determined by Bradford Reagent (Sigma-Aldrich), according to the manufacturer’s instructions. A total of 50 μg of proteins per sample in Laemmli Buffer 4× were run in a pre-cast gel (Bolt 4 to 12%, Bis–Tris, Invitrogen) until complete separation. In the case of murine-extracted samples, the tumors were collected at the same time point for the control and the treated animals, at day 35. The tumors were lysed in RIPA buffer and smashed using a pellet grinder. The protein amount was quantified as before, and 30 μg of proteins per sample in Laemmli Buffer 4× were run in 15% acrylamide gel. The proteins were then blotted on a polyvinylidene difluoride membrane, which was blocked in 5% skimmed milk and 0.1% Tween 20 in TBS for 2 h at room temperature and then treated with the appropriate primary (4 °C overnight) and secondary (2 h at room temperature) antibodies and revealed by chemiluminescence (ECL Bio-Rad). The antibodies used are listed in Supplementary Table 4. Densitometric analysis was performed using the free software ImageJ (for protein extracts from cells) and ImageLab by Bio-Rad (for protein extracts from mice samples) and protein expression levels were normalized to the level of either α-actinin or GAPDH.

### RT–qPCR

The total RNA content was extracted from cells treated for 48 h with 0.5 μM CK2-TN03 or with the carrier. Cells were previously counted and plated at a fixed amount (1,720,000 cells per 100 mm plate). The RNA was extracted using the phenol-chloroform method and its concentration was measured by Nano spectrophotometer. A total of 1 μg of RNA per sample was retrotranscribed using the Wonder RT cDNA synthesis kit (Euroclone) according to the manufacturer’s instructions. The obtained cDNA was diluted 1:5 and used for quantiative PCR with reverse transcription (RT–qPCR), performed on a Bio-Rad CFX96 thermocycler using the Excel-Taq FAST qPCR SybrGreen (Smobio) reagent, according to the manufacturer’s instructions. Every reaction was performed in triplicate and accompanied by the two negative controls consisting of water or non retrotranscribed RNA as template. The primers used are listed in Supplementary Table 5. The gene expression was normalized using the Pfaffl method with three different housekeeping genes.

### NCI-60 and ProLiFiler cell panel screenings

CK2-TN03 was first tested at two fixed doses (1 and 10 μM) in the NCI-60 panel (data not shown). Having satisfied the NCI-60 predetermined threshold inhibition criteria, the compound progressed to the five-dose screen. Details on the methodology are available at https://dtp.cancer.gov/discovery_development/nci-60/methodology.htm and in refs. ^37,38^.

Effect on viability was tested on the ProLiFiler (Reaction Biology Europe GmbH) panel. Compound treatment of cells started 1 day after seeding with a final DMSO concentration of 0.1%. DMSO 0.1% served as high control (100% viability) and staurosporine (1.0^−5^ M) as low control (0% viability). After incubation for 72 h at 37 °C, cell plates were equilibrated to room temperature for 1 h, CellTiterGlo reagent (Promega) was added and luminescence was measured approximately an hour later using a luminometer (EnVision, PerkinElmer). Raw data were converted into percent cell viability relative to the high and low control, which were set to 100% and 0%, respectively. EC_50_ calculation was performed using GraphPad Prism software with a variable slope sigmoidal response fitting model using 0% viability as the bottom constraint and 100% viability as the top constraint. Where the maximum effect leveled out at only partially reduced viability, the IC_50_ was calculated without bottom constraints.

Analyses were performed by Reaction Biology 4HF Biotec by correlating cell lines sensitivity to CK2-TN03 with those of 960 compounds included in the 4HF Biotec database^39^.

### Genotoxicity assay

The cytokinesis-block micronucleus assay was conducted as previously described^40,41^ and according to the Organization for Economic Co-operation and Development (OECD) guideline for the testing of chemicals (In Vitro Mammalian Cell Micronucleus Test No. 487).

### Cardiomyocytes differentiation and treatment

Induced pluripotent stem cells were grown using StemFlex medium (Gibco) and seeded on Geltrex coating in 12-well plates. At 80–90% confluency, the medium was changed into CardioMyocyte Differentiation medium, composed of RPMI 1640 (Gibco) with the addition of 2.5 mg/ml BSA (Merck Life Science), 200 μg/ml ascorbic acid, 1× GlutaMAX Supplement (Gibco) and 10 ng/ml FGF-2 (Peprotech). For the first 48 h, CHIR99021 (MedChemExpress) was added at a final concentration of 6 μM, followed by 48 h with Wnt-C59 (MedChemExpress) at 2 μM. The cells were then cultured in CardioMyocyte Differentiation medium for another 11 days, changing medium every 48 h. At day 13, CK2-TN03 at 0.5 μM or the vehicle was added to two or three wells (depending on the replicate). The treatment with CK2-TN03 lasted 48 h, after which 10% resazurin sodium salt solution (0.03 mg/ml powder from Sigma-Aldrich dissolved in PBS) was added directly into each well and incubated for 4 h at 37 °C, 5% CO_2_. The 570/600 nm fluorescence was measured using a Varioskan LUX (Thermo Scientific) plate reader. The viability was calculated as percentage compared with the vehicle-treated sample, considered 100% viable. A video of the beating cells was taken for each condition before and after the treatment, using a Leica DM IL LED microscope equipped with Leica dfc450c 0.70× at 10× magnification and the ScreenRec free software.

### In vivo treatment

(Ncr)-Foxn1 nu (nude mice) of 7–8 weeks were xenografted subcutaneously in the right flank with 7 × 10^6^ CHP-134 NB luciferase expressing cells in a 150 μl mixture of 1:1 cell medium: Matrigel (BD Bioscience). Approximately 15 days after injection, the tumor mass was palpable, and the mice were randomly assigned to either the control group treated with DMSO diluted to 10% or the treatment group treated with CK2-TN03 (40 mg/kg per day, *N* = 16). The drug administration was performed via ip injection once a day for a total of four weeks. Tumor progression was evaluated every week via live imaging (Bruker XTreme system) upon injection of D-luciferin (150 mg/kg, Sigma-Aldrich L9504) for the detection of luciferase signals. At the end of the treatment period, mice were monitored until the appearance of signs of tumor ulceration or tumor mass with a diameter equal or greater than 1.5 cm. At the reaching of this human end point, mice were killed by cervical dislocation. Tumor volume was calculated applying the modified ellipsoid formula 1/2(length × width^2^)^42^ based on the values obtained by in vivo imaging.

### Pharmacokinetics

All assays were conducted by Selvita SA.

Cell permeability and P-gp substrate assessment was performed in MDCKII-MDR1 cells. CK2-TN03 was tested in duplicate at 10 μM with amprenavir (0.5 μM) and diclofenac (10 μM) as controls. After incubation for 1 h, compounds were quantified by liquid chromatography with tandem mass spectrometry (LC–MS/MS) using warfarin as internal standard.

Protein binding was evaluated in mouse plasma by equilibrium dialysis. Compounds were tested in duplicate at 5 μM with an incubation time of 4 h and quantified by LC–MS/MS using diclofenac as internal standard. Nicardapine, verapamil and caffeine were used as control compounds.

Metabolic stability was checked in mouse liver microsomes (Corning-BD Biosciences). Compounds were tested in duplicate at 1 μM with sampling times 0, 10, 20, 30, 45 and 60 min and quantified by LC–MS/MS using diclofenac as internal standard. Testosterone, propranolol and caffeine were used as control compounds.

For in vivo pharmacokinetic studies, CK2-TN03 was dissolved in DMSO/HP-β-CD 40% in water (30/70), to reach final concentration of 2 mg/ml for an intravenous dose of 10 mg/kg (volume of administration 5 ml/kg). Three male CD-1 mice 6 weeks old (Charles River) were dosed into the lateral tail vein. Aliquots of blood were collected by serial (lateral tail vein) and terminal sampling (jugular vein). Samples were analyzed on a SCIEX triple quadrupole mass spectrometer operating in TurboIonSpray mode. Blank mouse plasma was used for preparation of blank samples, calibrations and quality control samples.

### Animal study approval

The experiments involving animals were conducted respecting ethical guidelines and were approved by the OPBA of the University of Trento and by the Italian Ministry of Health (authorization no.145/2022-PR).

## Results

### Selected compounds assume opposite poses in the CK2α ATP-binding pocket

Vinylguaiacol-carrying compounds were reported as potent and selective CK2 inhibitors^24^, as well as headgroups showing cross-reactivity with BET bromodomains^43^. To fully explore the inhibitor potential for this class of compounds and taking advantage of the ChEMBL database, the three most potent CK2 inhibitors bearing a vinylguaiacol moiety were selected (Table 1). In compound CK2-TN01, the terminal vinyl carbon carries a cyano group, which we already reported as a favorable substituent in the same position^24^, and a 5-amino-1H-pyrazole-4-carbonitrile linked via its C3. Compounds CK2-TN02 and CK2-TN03 are similar, bearing a 1,3-thiazolidine-2,4-dione and a 2-(phenylimino)-1,3-thiazolidin-4-one, respectively, with the remarkable difference of having the vinyl group in *para* (CK2-TN02, as in CK2-TN01) or *meta* (CK2-TN03) to the guaiacol hydroxyl. Compounds were tested by kinase assay on the catalytic CK2α subunit, confirming the same trend reported in the ChEMBL database (Table 1 and Supplementary Fig. 3).

**Fig. 1:**
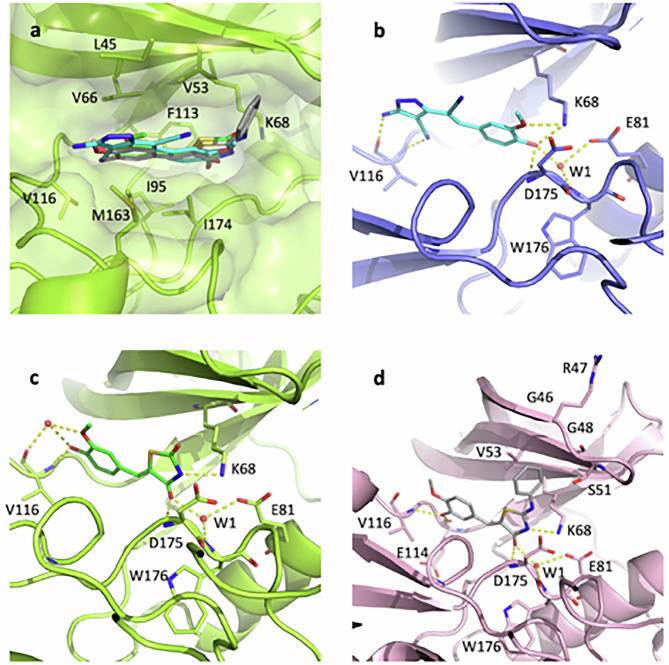
Crystallographic poses of the three inhibitors in the CK2 pocket. **a** Superposition of the three structures; CK2-TN01 is shown in cyan, CK2-TN02 in green and CK2-TN03 in gray. **b**–**d** Binding poses for each compound with hydrogen bonds shown as yellow dashed lines; the color code is the same as above (CK2-TN01 in panel **b**, CK2-TN02 in panel **c** and CK2-TN03 in panel **d**).

The crystallographic structure was solved for each compound in complex with CK2α. All molecules bind in the ATP pocket through numerous hydrophobic and van der Waals interactions, being sandwiched between residues Leu45, Val53, Val66 and Phe113 on one side and Ile95, Val116, Met163 and Ile174 on the other (Fig. 1a). They also anchor through ionic and polar interactions to the CK2α basic region (recognition of phosphate from ATP) and hinge region (interaction with the adenine ATP), although differently.

CK2-TN01 phenolic oxygen (predominantly as a negatively charged phenolate in the context of the CK2 basic region) is in contact with side chain of Lys68, main-chain nitrogen of Asp175 and the conserved water W1, at the very bottom of the cavity, connecting it to Glu81 side chain and Trp176 main-chain nitrogen (Fig. 1b); the methoxy oxygen is also in H-bond contact with Lys68 side chain. On the other side, the pyrazole ring anchors to the hinge region through hydrogen bonds formed by the 5-amino group and the 4-carbonitrile with Val116 main-chain oxygen and nitrogen, respectively.

CK2-TN02 and CK2-TN03 bind with opposite orientation with respect to CK2-TN01 (Fig. 1c, d). The thiazolidine-dione nitrogen of CK2-TN02 (deprotonated and negatively charged in the context of the CK2 basic region) and the thiazolidinone nitrogen of CK2-TN03 (also deprotonated and not assuming the imine tautomeric form, as evaluated by molecular docking) (Supplementary Table 6 and Supplementary Fig. 4) are in contact with Lys68 side chain, while the carbonyl oxygen on the same ring, common to both compounds, interacts with W1 and Asp175 main-chain nitrogen. On the same side of the ATP pocket, the extra phenyl ring of CK2-TN03 mainly stacks against Val53 side chain, being also in van der Waals contact with Ser51 side chain and with main chains of ^46^GRG^48^ from the glycine-rich loop (Fig. 1d). In CK2-TN02 the guaiacol oxygen interacts with Val116 main-chain nitrogen; moreover, a water molecule bridges both guaiacol oxygens to Val116 carbonyl (Fig. 1c). In CK2-TN03, hydroxyl and methoxyl groups are inverted with respect to CK2-TN02; the hydroxyl group forms hydrogen bonds to Glu114 main-chain oxygen and Val116 main-chain nitrogen, while the methoxyl group is kept closer and almost parallel to the hinge region (Fig. 1d).

The opposite orientations observed in CK2-TN02 and CK2-TN03 with respect to compound CK2-TN01 (structurally related compounds binding to the CK2 pocket in opposite orientations have been reported before^43^) are imposed by the CK2 basic region, which requires the most acidic atom (the phenolic oxygen in CK2-TN01 and the thiazolidine nitrogen in the others) to be located in contact with Lys68^43,44^ (Supplementary Fig. 5). This interaction is also relevant in defining the binding affinities, which correlate to the pKa of the inhibitors, with negatively charged compounds favored in their interaction with the positively charged region of CK2 (Table 1).

### CK2-TN03 causes dramatic cell death in NB cells

**Fig. 2:**
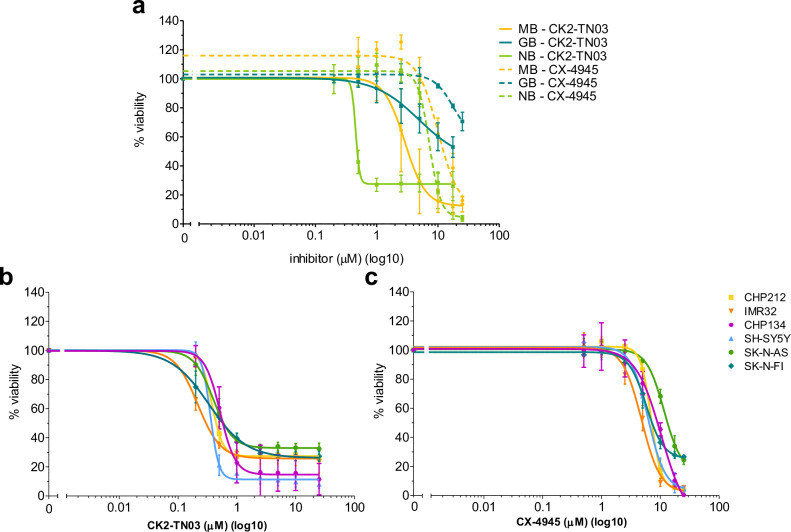
Effect of CK2-TN03 and CX-4945 on cellular viability. **a** Cell viability of MB cell line DAOY (yellow), GB cell line U87 (blue) and NB cell line CHP-212 (green), measured by Alamar blue assay, after 48 h exposure to increasing concentrations of CK2-TN03 (0.2–25 μM, straight lines) and CX-4945 (0.5–25 μM, dotted lines). **b**, **c** Cellular viability of six different NB lines exposed for 48 h to increasing concentrations of CK2-TN03 (0.2–25 μM) (**b**) or CX-4945 (0.5 to 25 μM) (**c**). Each point represents the mean ± s.d. of three to five independent experiments, depending on the cell line.

The therapeutic potential of CK2 inhibition in MB has been recently demonstrated^10,11^. The three inhibitors were then tested in cancer cell lines of neuroepithelial origin, namely glioblastoma (GB) U87, MB DAOY and NB CHP-212 cells in comparison with CX-4945. CK2-TN01 did not affect viability of all cell lines and CK2-TN02 was only barely effective at the maximum concentration tested (25 μM, Supplementary Fig. 6). Both CK2-TN03 and CX-4945 were active on GB cells at relatively high concentrations. Instead, they dramatically induced cell death in MB and even more in NB cell lines (Table 2 and Fig. 2a). In both cell lines, CK2-TN03 performed notably better than CX-4945 (about 6× and 15× lower EC_50_s in MB and NB, respectively), although being much less potent in kinase assay. The negative charge on the most acidic compounds, while increasing the affinity for CK2, may impact their cellular intake, reducing cellular efficacy.

**Table 2:** EC_50_ (μM) of CK2-TN03 and CX-4945 for MB, GB and NB cell lines.

	CK2-TN03	CX-4945
MB (DAOY)	1.95 ± 0.86	11.1 ± 2.9
GB (U87)	>25	>25
NB (CHP-212)	0.48 ± 0.07	7.9 ± 1.2
NB (CHP-134)	0.56 ± 0.17	9.2 ± 1.5
NB (IMR-32)	0.31 ± 0.08	5.1 ± 1.3
NB (SH-SY5Y)	0.41 ± 0.16	7.2 ± 2.0
NB (SK-N-AS)	0.55 ± 0.23	13.6 ± 3.7
NB (SK-N-FI)	0.58 ± 0.05	7.4 ± 1.5

The observed sensitivity of NB and MB cells to CK2 inhibition could be due to MYCN. NB is often a MYCN-driven cancer and MYCN is a known downstream target of the SHH pathway, overexpressed and/or amplified in SHH MB. CK2-TN03 and CX-4945 were then tested in five additional NB cell lines recapitulating, together with CHP-212, the most common alterations found in NB (Supplementary Table 7). Interestingly, all cell types were almost equally susceptible to CK2-TN03 or CX-4945, independently of their different genetic backgrounds (Table 2 and Fig. 2b, c), with CK2-TN03 consistently more effective than CX-4945. The observed efficacy of CK2 inhibition in the tested cells, either MYCN-amplified or not, suggests that cell death caused by CK2-TN03 and CX-4945 cannot be ascribed solely to the interference with the MYCN pathway. We notice that the only genetic alteration common to all tested cell lines is the partial 17q gain q21.31-qter.

### CK2-TN03 stalls NB cells in mitosis and promotes apoptosis

**Fig. 3:**
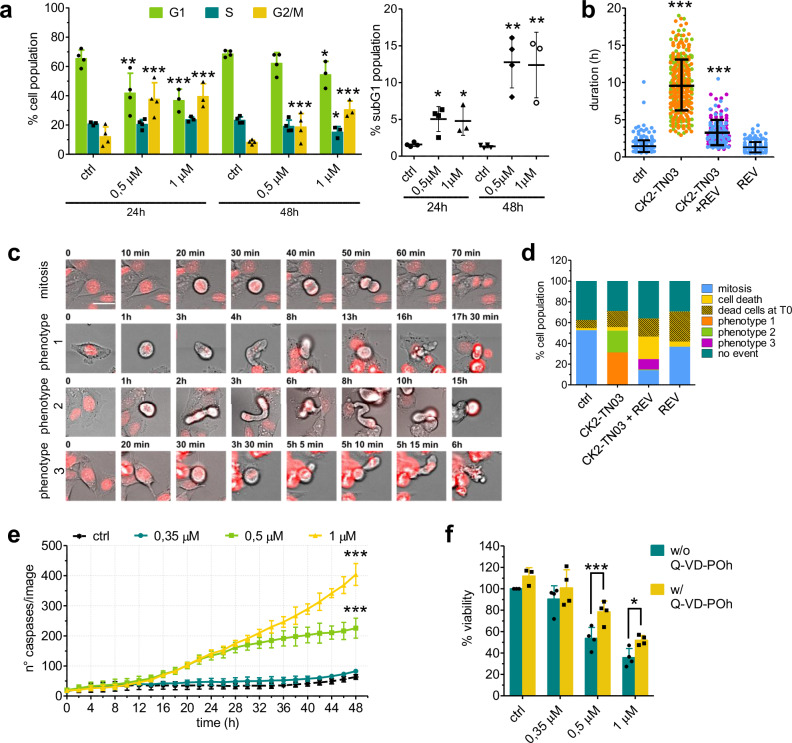
Effect of CK2-TN03 on cell cycle and apoptosis. **a** Percentage of CHP-212 cells in the cell cycle phases (left) and in the subG1 population of dead cells (right) after 24 or 48 h of treatment with 0.5 or 1 μM of CK2-TN03. Percentages for cell cycle phases only refer to dividing cells, while the percentage of cells in subG1 was calculated on the total number of analyzed cells. The bars represent the mean ± s.d. of four independent experiments. ns, *P* > 0.05; *P < 0.05; **P < 0.01; ***P < 0.001; one-way analysis of variance (ANOVA), Dunnett’s multiple comparison test, one-way ANOVA, comparing the percentage of cells in each phase of the cell cycle at each time point to the respective control. **b** Mean duration of the cellular events (mitosis or either phenotype caused by CK2-TN03). Time count started when the DNA condensed and the cell became rounded. Three different fields, each containing 200–300 cells, were analyzed for every condition. ns, *P* > 0.05; ***P < 0.001; one-way ANOVA, Dunnett’s multiple comparison test, one-way ANOVA, each duration compared only with the control sample. **c**, Timelapse images (scale bar, 20 μm) of CHP-212 cells treated with 0.5 μM CK2-TN03, with or without 0.2 μg/ml reversine. Pictures were taken every 5 min for 24 h, and the most representative pictures were selected to show the different phenotypes caused by the treatments. The DNA is shown in red, stained with siR-DNA. The first row shows normal mitosis in control samples; the second and third rows show phenotype 1 and 2, respectively, caused by 24 h treatment with 0.5 μM CK2-TN03; the fourth row shows phenotype 3, caused by the treatment with 0.5 μM CK2-TN03 combined with 0.2 μg/ml reversine. **d** Relative abundance of each event (normal mitosis, phenotype 1, 2 and 3, cell death) caused by the different treatments. The same cells considered for **c** were analyzed. **e** Number of cells with activated caspases 3/7, during a 48 h treatment with 0.35, 0.5 or 1 μM CK2-TN03. Cells, fluorescent green after caspase 3/7 activation, were automatically counted every 2 h by the Incucyte software, also used for imaging. Each point represents the mean ± s.d. of three independent experiments. ns, *P* > 0.05; ***P < 0.001 starting from hour 18 (showed only at the end of the curve, for clarity), two-way repeated-measures ANOVA, Bonferroni posttests, comparing the number of activated caspases at each treatment to the control. **f** Viability measured by Alamar Blue after 48 h treatment with 0.35, 0.5 or 1 μM CK2-TN03 in the presence (yellow) or absence (blue) of 10 μM pan-caspases inhibitor Q-VD-OPh. Each point represents the mean ± s.d. of four independent experiments. ns, *P* > 0.05; *P < 0.05, ***P < 0.001, two-way repeated-measures ANOVA, Bonferroni posttests, comparing viability calculated as percentage to control without and with Q-VD-OPh for each CK2-TN03 concentration.

Treatment of CHP-212 NB cells with CK2-TN03 significantly increased the number of tetraploid cells, as determined by FACS (Fig. 3a), indicating a cell cycle block in G2 or M phase. To spatially and temporally refine the observed effect, treated cells were monitored in timelapse over 24 h. CK2-TN03 caused a dramatic lengthening of the mitotic process impeding its completion (Fig. 3b). Two phenotypes appear and fully replace cells undergoing successful mitosis in vehicle-treated control (Fig. 3c, d). A first population goes through prophase and metaphase but does not enter anaphase. DNA remains concentrated in the middle of the cell that also starts producing elongated and dynamic cytosolic protrusions (Fig. 3c and Supplementary Movie 1). After 15–18 h, the elongated structures collapse and cells die. A second population is instead able to reach telophase with separated daughter sets of chromosomes, but cytokinesis fails. Cells again form cytosolic extrusions dying after a lengthy and erratic reshaping (Fig. 3c and Supplementary Movie 2).

The first phenotype could be determined by improper spindle organization, with the SAC (Spindle Assembly Checkpoint) coming into play and preventing cells from progressing to anaphase; loss of CK2 activity was reported to trigger SAC activation^45^. The second phenotype appears instead connected to missed telophase/cytokinesis coordination, possibly caused by malfunctioning CPC (Chromosomal Passenger Complex). Indeed, cotreatment of CHP-212 cells with CK2-TN03 and reversine, a SAC inhibitor, reversed the observed phenotype (Fig. 3b, d and Supplementary Movies 3 and 4). A fraction of cells is able to complete mitosis, while the remaining part undergoing duplication only briefly stalls in mitosis before going to apoptosis.

To verify the involved apoptotic machinery, caspases 3/7 activation induced by CK2-TN03 treatment was monitored by in-cell cleavage of a substrate peptide. Caspases activity progressively increased over time, suggesting the promotion of a caspase-dependent apoptotic mechanism (Fig. 3e). Indeed, cotreatment of CHP-212 cells with CK2-TN03 and Q-VD-OPh, a pan-caspase inhibitor, significantly reduced cell death (Fig. 3f); nonetheless, a relevant fraction of cells still dies pointing to the concomitant activation of a caspase-independent mechanism.

### CK2-TN03 counteracts survivin overexpression in NB cells

To elucidate the molecular mechanism underlying NB cell death induced by CK2-TN03, the q21.31-qter gain in chromosome 17, common to all NB cell lines tested, was scanned looking for genes regulating mitosis and apoptosis. Survivin emerged as the most obvious candidate, with all the previously described experiments pointing at it.

Survivin levels correlated with cancer stage in NB^46^. Its expression peaks at mitosis with >40-fold upregulation before being degraded at interphase. It is essential in orchestrating all mitotic phases being present in two different functional pools^47,48^. The first is associated with microtubules and involved in the assembly of the bipolar mitotic spindle. The second coordinates mitosis and cytokinesis by targeting the CPC (of which survivin is part) to the centromeres during anaphase and subsequently defining the cleavage plane for cellular division^49–51^.

Survivin is a member of the inhibitor of apoptosis family of proteins; it inhibits both intrinsic and extrinsic apoptotic pathways by recruiting other inhibitor of apoptosis members to the effector caspases 3 and 7. Accordingly, survivin depletion causes cytokinesis failure, mitotic catastrophe and apoptosis in various model systems, including NB^50–53^.

**Fig. 4:**
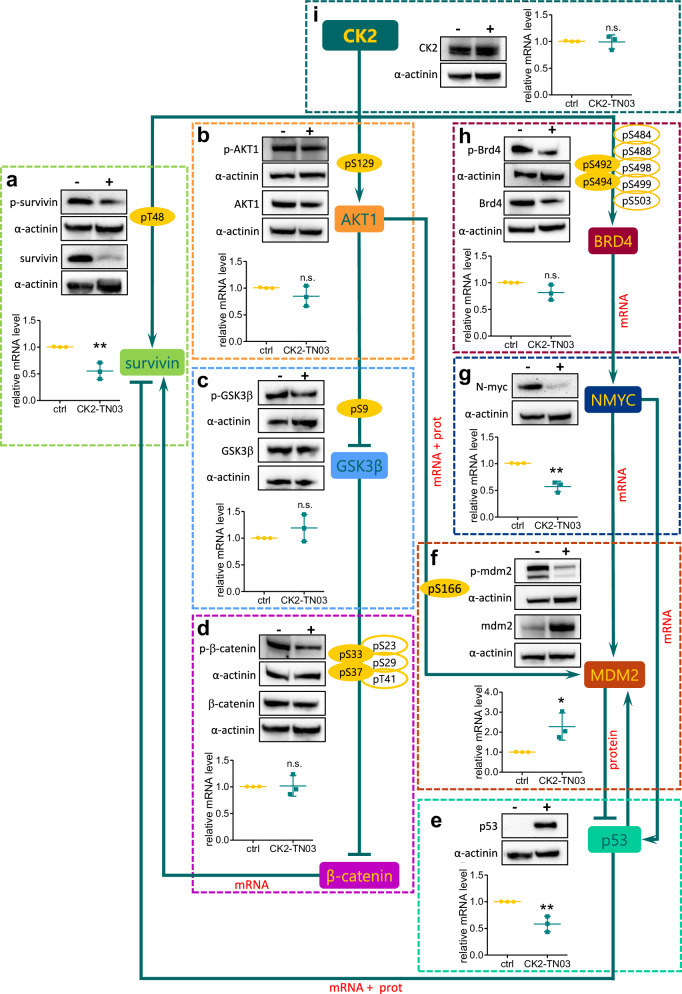
Schematic representation of CK2 downstream pathway affected by CK2-TN03. Western blots of total and phosphorylated protein levels (residue is indicated in the image) of the downstream effectors of CK2 analyzed in this work. **a**–**i**, Graphs showing the relative mRNA levels for all the proteins analyzed: survivin and phospho-survivin (T48) (**a**), AKT1 and phospho-AKT1(S129) (**b**), GSK3β and phospho-GSK3β(S9) (**c**), β-catenin and phospho-β-catenin(S33/37) (**d**), p53 (**e**), MDM2 and phospho-MDM2(S166) (**f**), MYCN (**g**), BRD4 and phospho-BRD4(S492/494) (**h**) and CK2 (**i**). A representative image is shown out of three to five independent experiments. The proteins were extracted from CHP-212 untreated cells (−) or cells treated for 48 h with 0.5 µM CK2-TN03 (+). The RNA was extracted from untreated cells or cells treated for 48 h with 0.5 µM CK2-TN03. The graphs show the mean ± s.d. of three independent experiments. ns, *P* > 0.05 (AKT1 *P* = 0.2251; GSK3β *P* = 0.2658; β-catenin *P* = 0.9095; Brd4 *P* = 0.0843; CK2 *P* = 0.8718); *P < 0.05 (MDM2 *P* = 0.0312); **P < 0.01 (survivin *P* = 0.0072; p53 *P* = 0.0076; MYCN *P* = 0.0016), two-tailed *t*-test.

Finally, survivin is regulated by CK2 by direct activation at the post-translational level^54^ and indirectly through transcriptional modulation^55,56^. In particular, CK2 activates survivin transcription through the AKT1/GSK3β/β-catenin axis, while removing its transcriptional inhibition through the BRD4/MYCN/MDM2/p53 pathway (Fig. 4).

Treatment with CK2-TN03 resulted in reduced survivin levels both in terms of mRNA and protein (−40% for both), with pThr48-survivin halved with respect to control vehicle-treated cells (Fig. 4a and Supplementary Fig. 7a). Activation of survivin transcription through β-catenin is not inhibited. Although AKT1 phosphorylation is reduced by about 30% (Fig. 4b and Supplementary Fig. 7b), pSer9-GSK3β is unaffected (Fig. 4c and Supplementary Fig. 7c), most probably because the same residue is also phosphorylated by RPS6KA3 and SGK3 in a compensatory manner^57,58^. Consequently, Ser33 and Ser37 phosphorylation of β-catenin, addressing it to proteasomal degradation, is minimally affected, and β-catenin mRNA and protein levels are largely unaffected (Fig. 4d and Supplementary Fig. 7d). We conclude that the observed reduction in survivin expression does not derive from the GSK3β/β-catenin axis.

Survivin levels are instead reduced by p53, which acts as a transcriptional repressor binding to the survivin promoter^59,60^. p53 protein levels are increased >25-fold by CK2-TN03 treatment, despite a significant reduction of its mRNA (Fig. 4e and Supplementary Fig. 7e). Increased p53 protein levels are caused by missing MDM2 activation, which requires phosphorylation by AKT1 to recognize and ubiquitinate p53^61^. Indeed, pSer166-MDM2 is reduced by 70%, despite doubled mRNA and protein levels (Fig. 4f and Supplementary Fig. 7f). Total MDM2 rise is dictated by p53, which promotes the expression of its own degrader in an autoregulatory loop^62^, while decreased p53 transcription can be ascribed to reduced MYCN levels^63,64^ (Fig. 4g and Supplementary Fig. 7g). MYCN transcription is dependent on BRD4 activation by CK2 phosphorylation^65–67^, which is consistently affected by CK2-TN03 (Fig. 4h and Supplementary Fig. 7h). We notice that the observed sensitivity to CK2-TN03 of NB cells, irrespective of their MYCN amplification status, is fully consistent with the proposed mechanism of action. Indeed, the reduced transcription of MDM2 and p53, induced by the lower level of MYCN in CK2-TN03 treated cells, is fully counteracted by the missing MDM2 activation and p53 degradation and their autoregulatory cycle. CK2 protein and mRNA levels are not affected by CK2-TN03 (Fig. 4i and Supplementary Fig. 7i).

In conclusion, we attribute the CK2-TN03 efficacy in NB cells to its overwhelming role with respect to survivin overexpression, exerted through diminished survivin phosphorylation and activation and reduced survivin expression obtained by alteration of the AKT1/MDM2/p53 axis. Low survivin activity then causes mitotic failure and apoptosis. Considering the CK2 generous substrate repertoire and the central role of p53 in determining the cell fate, we do not exclude that ancillary pathways come into play following CK2-TN03 treatment. Similar considerations apply to CK2-TN03 cross-reactivity on DAPK and PIM kinases. Indeed, when tested on a panel of 345 human kinases (Supplementary Table 8), CK2-TN03 also inhibited DAPK1, DAPK2, DAPK3, PIM1 and PIM3, with an improved selectivity profile with respect to CX-4945 inhibiting DAPK, PIM, CLK, HYPK and DYRK kinases, other than TBK1^68,69^. As PIMs have antiapoptotic functions, their inhibition could contribute to the observed CK2-TN03 efficacy; on the contrary, DAPKs are proapoptotic kinases.

### CK2-TN03 does not affect viability of quiescent cells nor is cardiotoxic and genotoxic

While abundant in various cancers, in adults survivin is only expressed in proliferating cells but undetectable in terminally differentiated tissues^70^; it is also expressed in pluripotent stem cells (and cancer stem cells) with its level gradually and rapidly decreasing in correspondence with the different stages of the in vitro differentiation^71,72^.

**Fig. 5:**
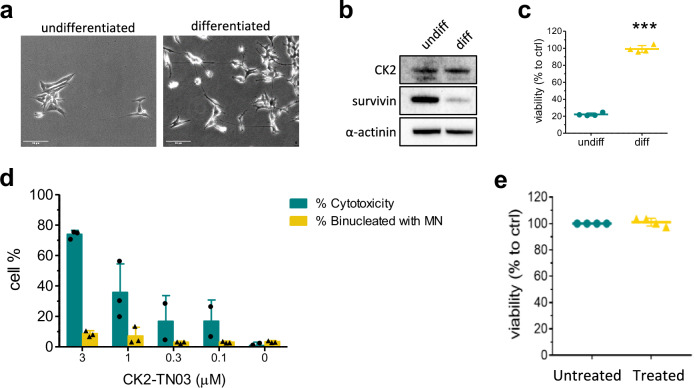
Effect of CK2-TN03 on differentiated NB cells and evaluation of genotoxicity and cardiotoxicity. **a** SH-SY5Y NB cells were differentiated for 5 days with 10 μM RA, followed by 4 days with 50 ng/μl BDNF, for a total of 9 days of differentiation. Scale bar, 50 μm. **b** At the end of the differentiation procedure, proteins were extracted from the SH-SY5Y cells and probed for the level of CK2 and survivin by western blot analysis, comparing the level of the two proteins to the one obtained in undifferentiated cells. **c** The viability of differentiated cells treated for 48 h with 0.5 μM CK2-TN03 was compared with the viability of actively replicating cells treated in the same fashion. Viability was measured by Alamar Blue assay and is shown as percentage compared with untreated cells for both conditions (viability of controls is not shown). The graphs show the mean ± s.d. of four independent experiments. ***P < 0.0001, two-tailed *t*-test. **d** Genotoxi**c**ity in CHO-K1 cell line caused by increasing concentration of CK2-TN03 is denoted by the number of micronuclei (MN) in binucleated cells. The graph shows the percentage of MN binucleated cells compared with untreated cells and compares the genotoxicity with the general cytotoxicity caused by CK2-TN03. The bars are the mean ± s.d. of three independent experiments. ns, *P* > 0.05; **P < 0.01; ***P < 0.001; two-way repeated-measures analysis of variance, Bonferroni posttests, where the percentage of MN or cytotoxicity obtained treating the cells with different CK2-TN03 concentrations was compared with the ones of the untreated cells. **e** Viability of cardiomyocytes treated for 48 h with 0.5 μM CK2-TN03 was compared with the viability of untreated cells. Viability was measured by the Alamar Blue assay and is shown as percentage compared with untreated cells. The graphs show the mean ± s.d. of four independent experiments.

To further connect the observed CK2-TN03 effects to survivin levels and activity, we tested the compound on SH-SY5Y NB cells following their differentiation by treatment with RA and BDNF (Fig. 5a). Survivin expression is reduced to about 20% with respect to undifferentiated cells and CK2-TN03 is ineffective on differentiated cells (Fig. 5b, c).

We finally excluded CK2-TN03 genotoxicity by micronucleus assay on CHO-K1 cells (Fig. 5d) and its cardiotoxicity by testing the molecule in cardiomyocytes (Fig. 5e and Supplementary Movies 5–12).

### In vivo CK2-TN03 administration reduces xenograft growth

**Fig. 6:**
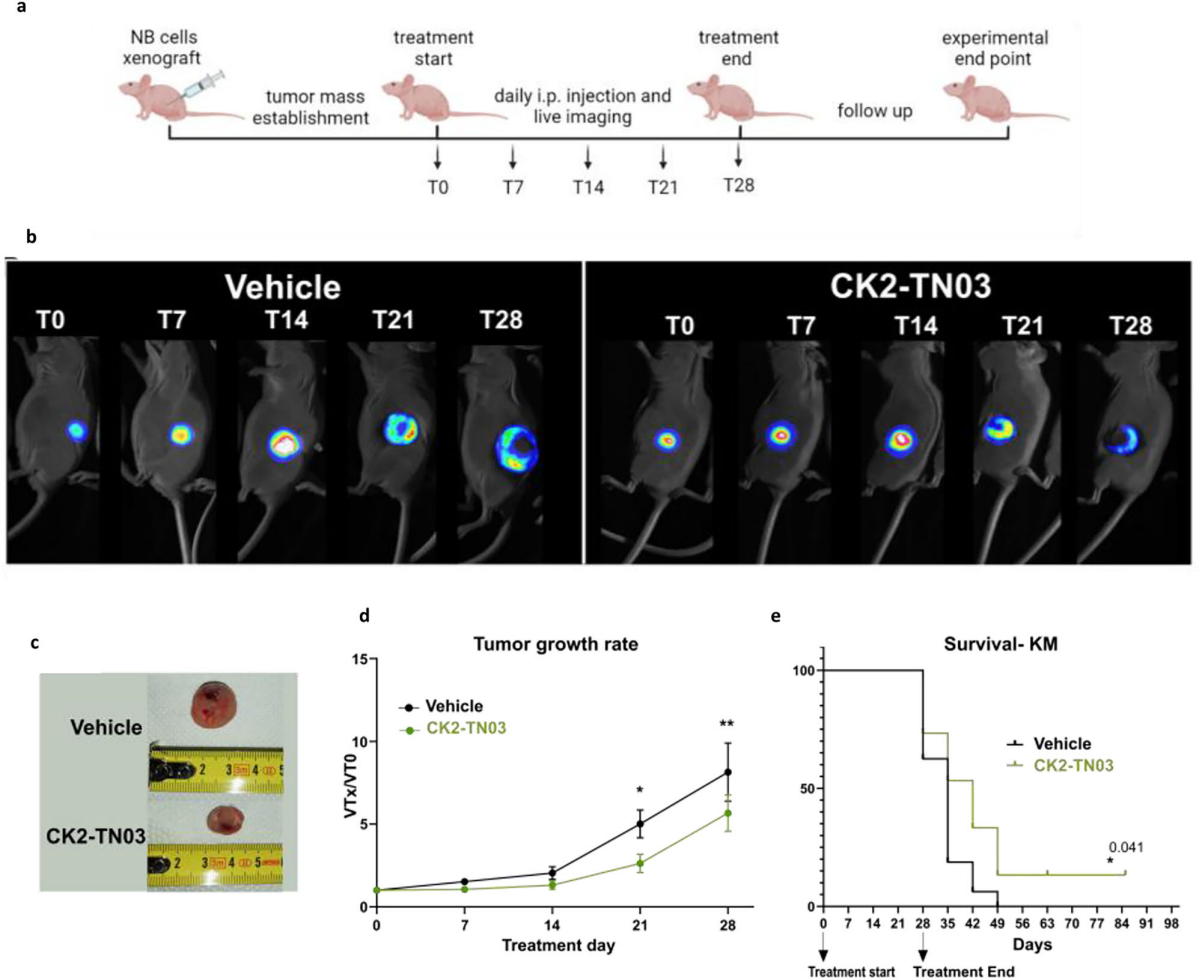
In vivo tumor growth is reduced by administration of CK2-TN03. **a** In vivo experiment timeline. **b** Representative images of tumor progression monitored by live imaging. **c** Images of mice xenografts extracted from the right flank after 28 days of vehicle or CK2-TN03 treatment. **d** Tumor growth rate in mice treated with either the vehicle (*N* = 16) or CK2-TN03 (*N* = 15). The data were normalized over tumor volume at time 0 h. Statistical significance was evaluated by multiple comparisons Fisher’s test. **e** Survival plot (KM) of differentially treated mice. The appearance of signs of tumor ulceration or diameter ≥1.5 cm was considered as the end point for mice killing. All statistically significant levels are reported as follows: *P < 0.05, **P < 0.01, ***P < 0.001.

The ability of CK2-TN03 to impact NB cells was subsequently evaluated in vivo. As mice model, we select (Ncr)-Foxn1 nu (nude mice) that were subcutaneously injected with CHP-134 cells. This cell line has been selected among the six lines previously tested in vitro, since it has well demonstrated engrafting and tumorigenic capacities in vivo. The treatment with either CK2-TN03 or vehicle was started just after the stabilization of the tumor mass, to mimic what occurs in the clinical practice and performed via intraperitoneal injection. The mice tumor masses were followed by in vivo imaging during the whole period of drug administration (4 weeks) (Fig. 6a). The overall-28days daily treatment with CK2-TN03 presents a notably different outcome with respect to administration of the vehicle (Fig. 6b). Looking at each time point separately, the CK2 inhibitor exposure leads to a slowdown of tumor growth resulting in the establishment of smaller tumors, especially after 21 and 28 days of administration (Fig. 6c, d). The hinder to tumor progression exerted by CK2-TN03 extended the overall survival probability of the mice; five mice treated with the inhibitor survived until 49 days from the start of the treatment protocol, while just one of the vehicle-treated mice reached this time point (Fig. 6e). Of note, two mice exposed to CK2-TN03 survived over to 85 days (Fig. 6e); a first one exerted complete remission showing no more luminescence signal at day 14, while the second one only presented a small quiescent mass that stopped growing even when treatment ended.

In conclusion, data clearly show that the in vivo administration of the CK2 inhibitor CK2-TN03 slows down tumor progression, negatively impacting its growth. Furthermore, the CK2-TN03 mechanism of action has been confirmed in the extracted tumor masses that show increased levels of p53 and decreased survivin levels and phosphorylation in the treated animals (Supplementary Fig. 8).

### Efficacy of CK2-TN03 on other cancer cell lines and inferred mechanism of action

**Fig. 7:**
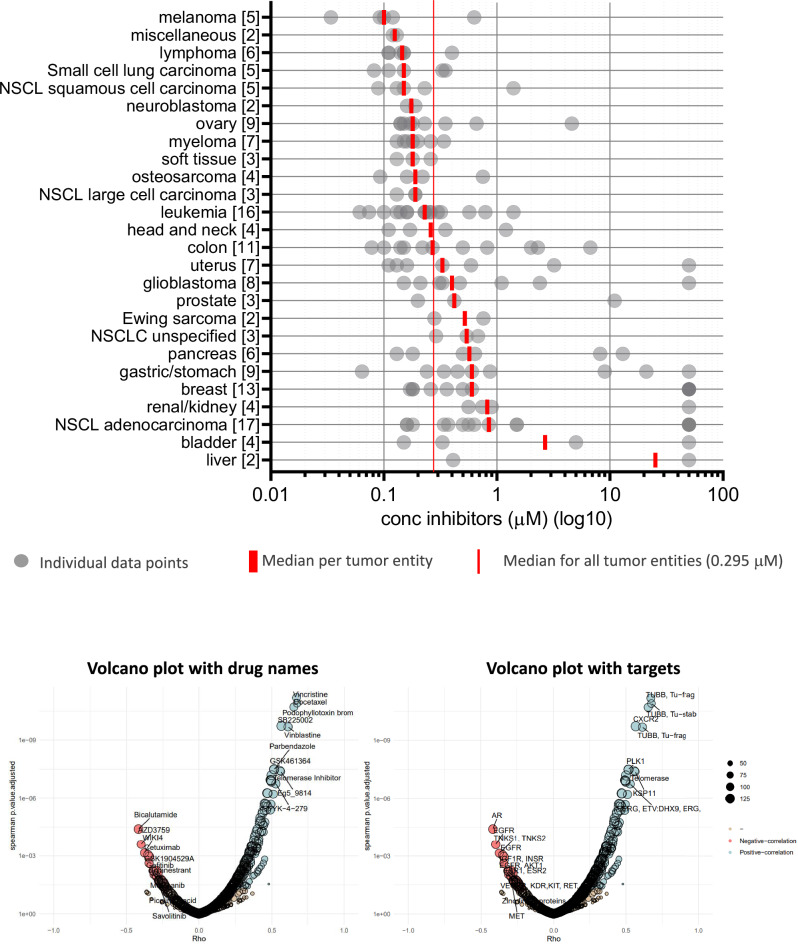
Efficacy profile of CK2-TN03 on 160 cell lines and correlation with drugs of known MoA. Top: ProLiFiler screen: medians per cancer entity vary from 0.1 to 25.2 µM (sorted from top to bottom by increasing values). A significant tumor entity selectivity (Kruskal–Wallis *P* value = 0.0005) is appreciable. Two cell lines classified as miscellaneous (HUTU-80, duodenal carcinoma and A431, epidermoid carcinoma) are also highly susceptible. Bottom: volcano plots correlating CK2-TN03 efficacy on cancer cell lines with 897 anti-cancer drugs; *P* values and rho coefficients for the top ten hits are reported in Supplementary Table 4.

CK2-TN03 was tested on two different cancer cell panels. The NCI-60 (National Cancer Institute, 60 cell lines) and the ProLiFiler (Reaction Biology, 160 cell lines) panels were interrogated. Although the two standardized testing protocols are different (that is, 48 versus 72 h and sulforhodamine B versus CellTiterGlo detection, respectively), the obtained GI_50_ (NCI-60, Supplementary Fig. 9) and EC_50_ (ProLiFiler, Supplementary Figs. 10 and 11) values correlate in the 24 cancer cell lines shared between the two panels. The ProLiFiler screens identified melanoma, blood cancers (lymphomas > myeloma > leukemia), soft tissues tumors, ovarian cancer and osteosarcoma as the most affected cancer entities, other than NB (Fig. 7). On the contrary, liver, bladder, kidney, breast, stomach and pancreas cancers are the least affected. For ovarian cancer, it is worth noting that the rare small-cell carcinoma and Brenner tumor cell lines (COV434 and SNU840) are less affected by CK2-TN03. Lung cancers are the most represented (32 cell lines), permitting their further dissection in subtypes. Indeed, small-cell lung cancer, squamous-cell carcinoma and large-cell carcinoma join the list of highly affected tumors, while NSCLC are among the least affected cancer types.

The ProLiFiler sensitivity profile of CK2-TN03 was then compared with those of drugs with known mechanism of action. Top hits all represent molecules affecting microtubule assembly, mitotic spindle formation and mitosis progression (Fig. 7 and Supplementary Table 9). These includes microtubule polymerization inhibitors (vincristine, vinblastine, docetaxel, parbendazole and podophyllotoxin), a kinesin inhibitor (affecting KIF11, required for establishing the bipolar spindle) and an inhibitor of PLK1 (regulating centrosome maturation, spindle assembly, mitotic exit and cytokinesis, also acting in concert with CK2^73^). Regarding the additional hits, the chemokine receptor inhibitor SB225002 has also been reported to induce mitotic catastrophe by downregulating Chk1 and activating Cdk1^74^, while the telomerase inhibitor IX (aka MST-312) and YK-4-279 (an inhibitor of ETS transcription factors) both induce mitotic defects. In particular, MST-312 has been shown to affect CPC functionality, also reducing survivin level^75^, while YK-4-279 inhibits the formation of kinetochore microtubules without affecting their acetylation as instead observed with paclitaxel and vincristine^76^. CK2-TN03 did not directly interfere with tubulin polymerization when tested in an in vitro polymerization assay at concentrations spanning the EC_50_ values measured in the ProLiFiler panel (Supplementary Fig. 12).

Although the correlation with the single drugs is moderate (Spearman’s *ρ* 0.52–0.68), the overall picture confirms CK2-TN03 phenotypic effects and affected cellular processes, that is, mitotic spindle and the CPC. In this context, it is worth noting that CK2 has been reported to promote microtubule polymerization and spindle assembly by various mechanisms^45,73,77–79^. The efficacy of CK2-TN03 on the various cancer cell lines is probably connected to the dependency of their proliferative potential from cancer-type specific alterations in those processes, for example, survivin overexpression in NB.

### Pharmacokinetic characterization

CK2-TN03 showed very good permeability and negligible efflux ratio, not being a P-gp substrate, in MDCKII-MDR1 cells (Supplementary Table 10). While the compound undergoes high in vitro clearance when tested in mouse liver microsomes (Supplementary Table 11 and Supplementary Fig. 13), it is also characterized by very high plasma protein binding (Supplementary Table 12), with this last that should limit in vivo clearance. Consistently, CK2-TN03 showed a steep decline in plasma concentration in vivo in the first hour associated with a large volume of distribution (Supplementary Table 13 and Supplementary Fig. 14); the compound is affected by a combination of metabolic clearance and rapid partitioning into tissues with the half-life (*t*_1/2_) = 12 min in the distribution phase. The remaining fraction is cleared much more slowly (t_1/2_ = 5 h in the elimination phase), remaining stable and quantifiable in plasma up to the experimental end point at 24 h.

The above data, for example, limited metabolic stability counteracted by very good permeability and volume of distribution, agree with the observed in vivo efficacy of CK2-TN03.

### Exploration of the CK2-TN03 chemical class

Taking advantage of the CK2-TN03 crystallographic pose, a library of 247 derivatives was designed and tested by molecular docking (Supplementary Fig. 15). Compounds were filtered for their immediate availability in commercial collections and thirteen were then selected based on computed docking scores (TN11-13, 15-22 and 25); TN14 and 23 were added as compounds with reduced polar surface area and predicted BBB permeability and TN24 for improved solubility (Supplementary Table 14). Compounds showing >50% reduction of cell viability when tested in single dose at 2.5 μM were evaluated in dose–response mode (Table 3 and Supplementary Fig. 16). Modifications on the guaiacol ring are generally poorly accepted with the exception of TN13 bearing the substitution methoxy to ethoxy group. Compounds with small hydrophobic substituent in ortho or meta of the aminophenyl group performed instead slightly better than CK2-TN03.

**Table 3:** EC_50_ (μM) of CK2-TN03 analogs in CHP-212 NB cell line.

	Structure	EC_50_ (μM)		Structure	EC_50_ (μM)
TN11		0.096 (±0.015)	TN12		0.106 (±0.019)
TN13		0.101 (±0.021)	TN15		0.785 (±0.063)
TN16		2.18 (±0.37)	TN17		0.904 (±0.036)
TN18		0.187 (±0.024)	TN19		0.094 (±0.023)
TN20		0.139 (±0.020)	TN25		0.275 (±0.042)

**Fig. 8:**
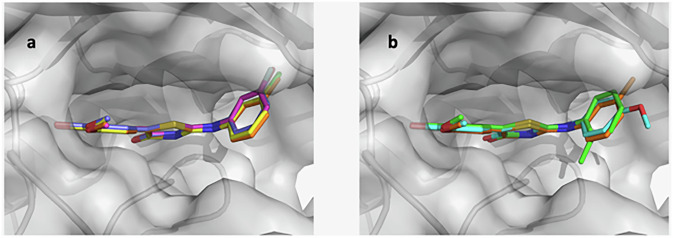
Crystallographic structures of CK2-TN03 analogs in the CK2 ATP-binding pocket. **a** Meta-substituted compounds assume identical poses, (TN12, TN16, TN19 and TN20 are shown in orange, slate, magenta and yellow, respectively). **b** Crystallographic poses for TN11, TN12 and TN17 are shown in green, orange and cyan, respectively.

We finally determined the crystallographic structures for six of the above compounds in complex with CK2 (Fig. 8). Compounds TN12, TN16, TN19 and TN20 bear four different meta-substituents on the aminophenyl group. They bind in the CK2 pocket with identical poses; the extra group (with respect to CK2-TN03) occupies a small cavity below the tip of the N-terminal β-sheet. While CK2-TN16 hydroxyl group establishes hydrogen bonds with Ser51, fluoro, chloro and methyl groups exploit various van der Waals contacts in this predominantly hydrophobic spot (Supplementary Fig. 5). Interestingly, the ortho methyl group in compound CK2-TN11 points in the opposite direction back toward the interior of the cavity, while the CK2-TN17 para methoxy is parallel to the N-terminal β-sheet and more solvent exposed.

## Discussion

**Fig. 9:**
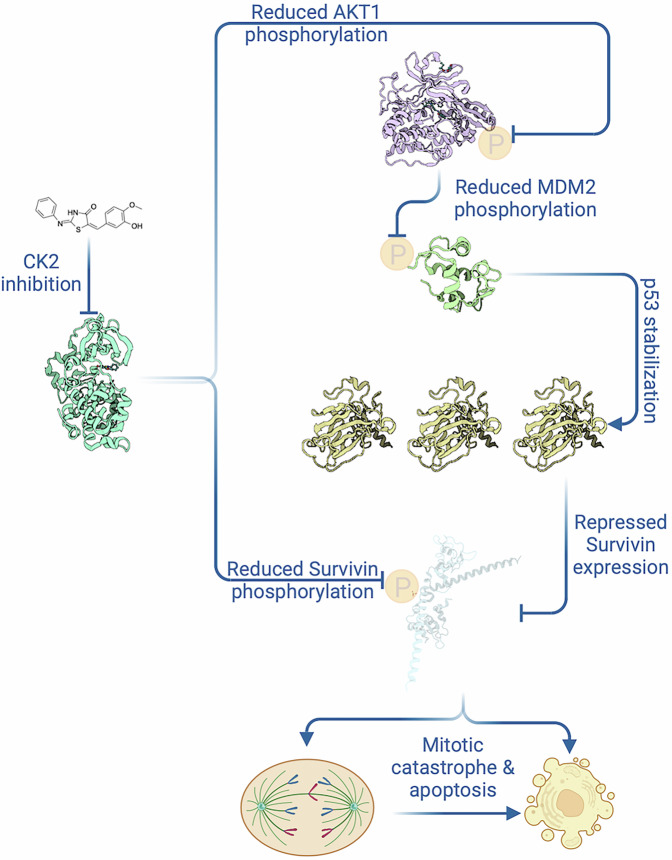
Mechanism of action for CK2-TN03. Mitotic catastrophe and apoptosis are determined by CK2 inhibition through survivin downregulation imposed both directly and via the AKT1/MDM2/p53 pathway. Created with BioRender.com (Lolli, G. (2025)).

All data presented above support the exploitation of CK2 inhibitors for NB treatment. In particular, we show that CK2-TN03 is an ATP-competitive CK2 inhibitor inducing death in many NB cell lines at nanomolar concentration and more effectively than the clinical grade inhibitor CX-4945, also being more selective on the human kinome. Its mechanism of action relies on the suppression of survivin, overexpressed in NB, both in terms of activity (inhibition of survivin phosphorylation by CK2) and protein level (repression of survivin transcription through stabilization of p53) (Fig. 9). Consistently with the above mechanism of action, CK2-TN03 causes prolonged cell cycle block in mitosis and activation of the apoptotic machinery in NB cells, while being ineffective on quiescent cells where survivin is barely expressed. When tested on a large panel of cancer cell lines, CK2-TN03 showed selectivity among different tumor types, with an efficacy profile resembling drugs interfering with mitotic processes. CK2-TN03 hinders tumor growth in mice xenografts without any apparent toxicity, with a combination of pharmacokinetics properties correlating with the observed in vivo efficacy and tractability of its chemical class permitting further optimization.

## Supplementary information

Supplementary Information

Timelapse movie of CHP-212 cells treated with CK2-TN03 (phenotype 1).

Timelapse movie of CHP-212 cells treated with CK2-TN03 (phenotype 2).

Timelapse movie of CHP-212 cells treated with CK2-TN03 and reversine.

Timelapse movie of untreated CHP-212 cells.

Untreated cardiomyocytes time 0 h replicate 1.

Untreated cardiomyocytes time 0 h replicate 2.

Treated cardiomyocytes time 0 h replicate 1.

Treated cardiomyocytes time 0 h replicate 2.

Untreated cardiomyocytes time 48 h replicate 1.

Untreated cardiomyocytes time 48 h replicate 2.

Treated cardiomyocytes time 48 h replicate 1.

Treated cardiomyocytes time 48 h replicate 2.

## Data Availability

CK2 structures were deposited to the PDB with accession numbers 8C6L (CK2-TN01, https://www.rcsb.org/structure/8C6L), 8C6M (CK2-TN02, https://www.rcsb.org/structure/8C6M), 8C6N (CK2-TN03, https://www.rcsb.org/structure/8C6N), 9I0Z (TN11, https://www.rcsb.org/structure/9I0Z), 9I10 (TN12, https://www.rcsb.org/structure/9I10), 9I11 (TN16, https://www.rcsb.org/structure/9I11), 9I12 (TN17, https://www.rcsb.org/structure/9I12), 9I13 (TN19, https://www.rcsb.org/structure/9I13) and 9I17 (TN20, https://www.rcsb.org/structure/9I17). Atomic coordinates and experimental data have been released.
