# Supplementary material for: Switching off CK2-mediated activation of survivin offers new therapeutic opportunities in neuroblastoma
Source: Exp Mol Med. 2026 Jan 22;58(1):227–42. doi: 10.1038/s12276-025-01628-5 (PMC12868639; doi:10.1038/s12276-025-01628-5)
Supplement: Supplementary file 1 — Supplementary Information [file 12276_2025_1628_MOESM1_ESM.pdf]

Switching off CK2-mediated activation of survivin offers new therapeutic opportunities in neuroblastoma

Giulia Cazzanelli[1*], Andrea Dalle Vedove[1], Francesca Broso[1], Matteo Burigotto[1], Jacopo Zasso[1], Giuseppe Aiello[1], Francesca Zonta[2], Andrea Astolfi[3], Maria Letizia Barreca[3], Maria Ruzzene[2], Luca Tiberi[1], Luca L. Fava[1], Alessandro Quattrone[1] and Graziano Lolli[1*]

Author Affiliations:

[1]Department of Cellular, Computational and Integrative Biology - CIBIO, University of Trento, via Sommarive 9, 38123 Povo - Trento, Italy

[2] Department of Biomedical Sciences and CNR Institute of Neuroscience, University of Padua, Via U. Bassi 58/B, 35131 Padua, Italy

[3]Department of Pharmaceutical Sciences, University of Perugia, via Fabretti 48, 06123 Perugia, Italy

* *Correspondence to Giulia Cazzanelli and Graziano Lolli*: giulia.cazzanelli@unitn.it; graziano.lolli@unitn.it.

Investigation of CK2-TN03 tautomerism by molecular docking

CK2-TN03 can exist in various tautomeric forms. We performed molecular docking simulations to determine which tautomer could exhibit a CK2 binding mode and binding affinity consistent with the experimental data. Indeed, the co-crystallographic structure revealed that the ligand's thiazolidinone nitrogen was in close proximity to the positively charged head of Lys68, suggesting the presence of a strong polar intermolecular interaction. In view of this observation, three tautomeric forms of CK2-TN03 were considered in our analysis (Supplementary Fig. 2), where the TN03-T2 tautomer was considered in its deprotonated state due to the possible favorable interaction between the protonated (cationic) residue Lys68 and the anionic nitrogen atom of the ligand.

In parallel, we evaluated the impact of the conserved water molecule W1 (Fig. 1 in the main text) on the docking predictions.

Thus, six different CK2-TN03 complexes (Supplementary Table 1) as described in the Methods section of the main text.

The docking results showed that all three explored tautomers were able to reproduce the ligand crystallographic orientation (RMSDs ≤ 0.5 Å). Notably, regardless of the protein model, the TN03-T2 tautomer always performed as the best-scored docking client (Supplementary

Table 1), especially when combined with the protein model including the conserved water molecule W1 (i.e., T2-1W model) (Supplementary Fig. 2).

Supplementary Table 1. Data Collection and Refinement Statistics

	CK2-TN01	CK2-TN02	CK2-TN03
Data Collection			
Space group	$P2_1$	$P2_1$	$P2_1$
Unit-cell parameters (Å, °)	a = 58.45<br>b = 46.36<br>c = 63.26<br>β = 112.02	a = 58.74<br>b = 46.29<br>c = 63.65<br>β = 111.85	a = 58.49<br>b = 46.52<br>c = 63.43<br>β = 111.84
Wavelength (Å)	1.000	1.000	1.000
Resolution (Å)	46.36-1.80 (1.84-1.80)	46.29-1.80 (1.84-1.80)	46.52-2.05 (2.11-2.05)
R_{merge} (%)	7.9 (72.4)	9.9 (74.9)	14.8 (83.1)
R_{meas} (%)	9.0 (82.5)	10.8 (80.9)	16.4 (92.1)
R_{pim} (%)	4.2 (39.0)	4.2 (30.4)	6.9 (38.9)
$<I/\sigma(I)>$	11.5 (2.1)	12.6 (2.6)	8.6 (2.2)
$CC^{1/2}$	0.997 (0.728)	0.997 (0.875)	0.995 (0.755)
Completeness (%)	99.9 (99.9)	99.9 (99.8)	99.8 (99.7)
Multiplicity	4.5 (4.4)	6.6 (6.9)	5.5 (5.4)
Refinement			
Resolution (Å)	36.38-1.80	36.44-1.80	36.51-2.05
R_{work}/R_{free} (%)	16.4/20.9	15.9/20.2	17.9/21.8
R.m.s. deviations			
Bond lengths (Å)	0.006	0.007	0.007
Bond angles (°)	0.80	0.87	0.94
PDB entry	8C6L	8C6M	8C6N

Supplementary Table 2. Data Collection and Refinement Statistics

	TN11	TN12	TN16
Data Collection			
Space group	$P2_1$	$P2_1$	$P2_1$
Unit-cell parameters (Å, °)	a = 58.41<br>b = 46.74<br>c = 63.38<br>β = 111.92	a = 58.35<br>b = 46.39<br>c = 63.36<br>β = 111.57	a = 58.48<br>b = 46.63<br>c = 63.30<br>β = 111.60
Wavelength (Å)	1.000	1.000	1.000
Resolution (Å)	58.80-1.55 (1.58-1.55)	58.92-1.50 (1.53-1.50)	58.86-1.60 (1.63-1.60)
R_{merge} (%)	5.6 (90.5)	5.8 (77.1)	4.3 (56.7)
R_{meas} (%)	6.1 (98.6)	6.3 (83.8)	4.7 (63.9)
R_{pim} (%)	2.4 (38.6)	2.5 (32.3)	1.9 (28.5)
$<I/\sigma(I)>$	18.1 (2.1)	16.2 (2.5)	19.3 (2.5)
$CC^{1/2}$	0.999 (0.799)	0.999 (0.850)	0.999 (0.796)
Completeness (%)	99.9 (100.0)	99.9 (100.0)	99.7 (98.5)
Multiplicity	6.4 (6.4)	6.4 (6.6)	6.1 (4.8)
Refinement			
Resolution (Å)	36.60-1.55	36.46-1.50	36.56-1.60
R_{work}/R_{free} (%)	16.9/19.4	16.3/19.3	16.4/19.6
R.m.s. deviations			
Bond lengths (Å)	0.006	0.006	0.006
Bond angles (°)	0.87	0.87	0.85

PDB entry	9I0Z	9I10	9I11

Supplementary Table 3. Data Collection and Refinement Statistics

	TN17	TN19	TN20
Data Collection			
Space group	$P2_1$	$P2_1$	$P2_1$
Unit-cell parameters (Å, °)	a = 58.08<br>b = 45.85<br>c = 62.61<br>β = 111.23	a = 58.49<br>b = 46.61<br>c = 63.41<br>β = 111.75	a = 58.40<br>b = 46.51<br>c = 63.31<br>β = 111.50
Wavelength (Å)	1.000	1.000	1.000
Resolution (Å)	58.36-2.00 (2.05-2.00)	58.90-1.75 (1.78-1.75)	58.91-1.55 (1.58-1.55)
R_{merge} (%)	8.8 (87.9)	4.9 (56.3)	6.1 (73.1)
R_{meas} (%)	9.7 (96.3)	5.3 (63.8)	6.6 (79.6)
R_{pim} (%)	4.0 (38.8)	2.1 (29.0)	2.6 (31.1)
$\langle I/\sigma(I) \rangle$	11.7 (2.3)	19.5 (2.6)	16.2 (2.7)
$CC^{1/2}$	0.998 (0.746)	0.999 (0.777)	0.999 (0.824)
Completeness (%)	99.9 (100.0)	99.1 (86.7)	99.9 (100.0)
Multiplicity	5.8 (5.9)	6.1 (4.6)	6.3 (6.4)
Refinement			
Resolution (Å)	49.67-2.00	36.56-1.75	36.51-1.55
R_{work}/R_{free} (%)	16.6/22.3	16.0/19.4	16.4/19.2
R.m.s. deviations			
Bond lengths (Å)	0.007	0.006	0.006
Bond angles (°)	0.86	0.86	0.84
PDB entry	9I12	9I13	9I17

Supplementary Table 4. Antibodies used for western blots

PROTEIN	BRAND	DILUTION
**primary**		
CK2	sc-12738 Santa Cruz Biotechnology	1:150
Survivin	sc-17779 Santa Cruz Biotechnology	1:250
Phospho-survivin (T48)	PA5-105388 Invitrogen	1:500
AKT1	sc-5298 Santa Cruz Biotechnology	1:200
Phospho-AKT1 (S129)	MAB20322 Abnova	1:300
GSK-3β	#9315 Cell Signaling Technology	1:1000
Phospho-GSK-3β (S9)	#9323 Cell Signaling Technology	1:1000
β-catenin	#8480 Cell Signaling Technology	1:1000
Phospho-β-catenin (S33/37)	#2009 Cell Signaling Technology	1:500
Brd4	ab128874 abcam	1:200
Phospho-Brd4 (S492/494)	#ABE1453 Millipore	1:350
N-myc	ab16898 abcam	1:200
Mdm2	#86934 Cell Signaling Technology	1:1000
Phospho-MDM2 (S166)	ab170880 abcam	1:50000
p53	sc-123 Santa Cruz Biotechnology	1:500
α-actinin	sc-17829 Santa Cruz Biotechnology	1:4000
GAPDH	GTX100118 GeneTex	1:20000
**secondary**		
m-IgGκ BP-HRP	sc-516102 Santa Cruz Biotechnology	1:1500
Mouse anti-rabbit	sc-2357 Santa Cruz Biotechnology	1:1500
Goat Anti-Rabbit IgG (H&L) -HRP Conjugated	CSA2115 Cohesion Bioscience	1:10000
Goat anti-mouse HRP	62-6520 Invitrogen	1:4000

Supplementary Table 5. Primers used for RT-qPCR experiments

gene		sequence
CK2	FW	CGAGTTGCTTCCCGATACTTC
	RV	ACTTGCCAGCATACAACCCAA
P53	FW	CGCTTCGAGATGTTCCGAGA
	RV	TTTGGACTTCAGGTGGCTGG
NMYC	FW	GACACCCTGAGCGATTCAGATGAT
	RV	GGTGAATGTGGTGACAGCCT
MDM2	FW	GGCAGGGGAGAGTGATACAGA
	RV	GAAGCCAATTCTCACGAAGGG
BIRC5 (Survivin)	FW	ATGACGACCCCATAGAGGAAC
	RV	CGCACTTTCTCCGCAGTTTC
AKT1	FW	ACTGTCATCGAACGCACCTT
	RV	AAACTCGTTCATGGTCACGC
BRD4	FW	GAGCTACCCACAGAAGAAACC
	RV	GAGTCGATGCTTGAGTTGTGTT
GSK3B	FW	AGACGCTCCCTGTGATTTATGT
	RV	CCGATGGCAGATTCCAAAGG
CTNNB (β- catenin)	FW	GGAAGGTCTGAGGAGCAGCTT
	RV	GTCCAACTCCATCAAATCAGCTTG
Housekeeping genes		
HPRT1	FW	TGACACTGGCAAAACAATGCA
	RV	GGTCCTTTTCACCAGCAAGCT
B2M	FW	TGCTGTCTCCATGTTTGATGTATCT
	RV	TCTCTGCTCCCCACCTCTAAGT
ACTB	FW	TGTACGCCAACACAGTGCTG
	RV	GCTGGAAGGTGGACAGCGA

Supplementary Table 6. Docking score (in Kcal/mol) computed for the three tautomeric forms of CK2-TN03 (i.e. T1-3) docked into the six CK2 protein models

	Protein model					
	**T1-noW[a]**	**T1-1W[a]**	**T2-noW[a]**	**T2-1W[a]**	**T3-noW[a]**	**T3-1W[a]**
**TN03 -T1**	-8.745	-8.934	-8.79	-9.036	-8.838	-8.693
**TN03 -T2**	-9.185	-9.799	-10.009	-10.022	-9.066	-9.773
**TN03 -T3**	-9.116	-9.586	-8.918	-9.776	-8.853	-9.592

[a] The protein models are indicated considering the ligand tautomeric form used to prepare the receptor (i.e. T1-3) and the presence (-W1) or absence (-noW) of the conserved water molecule.

Supplementary Table 7: comparison between genetic alteration of the different neuroblastoma cell lines used in this study (adapted from Harenza 2017)

	MYCN amplification	17q gain	Deletion 1p31-term	LOH at 11q	p53 status	Corresponding tumour type (Łastowska 2001)
**CHP-212**	yes	Gain q12-qter	Loss 1p13.2-pter	LOH 11q23.3	wt	Type 3
**CHP-134**	yes	Gain q12-qter	LOH p32.3-pter; Gain p34.3-p36.22; Loss p36.22-pter	no	wt	Type 3
**IMR-32**	yes	Gain q21.2-ter	Loss p32.3-pter	cnLOH q23.1	wt	Type 3
**SH-SY5Y**	no	Gain q21.31-qter	no	Loss q22.1-24.2	wt	Type 2 (group 2)
**SK-N-AS**	no	Gain q21.31-qter	Loss p36.22-36.32	Loss q13.1-qter	H168R p53β isoform	Type 2 (group 3)
**SK-N-FI**	no	Gain q21.31-qter	no	no	M246R	Type 2 (group 2)

Supplementary Table 8. Residual activity values for CK2-TN03 in 345 wild-type protein kinase assays. All values are calculated as % of control.

Residual activity < 50 %

			Cpd ID	CK2-TN03	CK2-TN03
#	Kinase Name	Kinase Family*	Assay Conc. (M)	8,25E-07	1,65E-06
1	ABL1	TK		92	89
2	ABL2	TK		105	95
3	ACK1	TK		94	89
4	ACVR1	TKL		95	94
5	ACVR1B	TKL		97	95
6	ACVR2A	TKL		99	104
7	ACVR2B	TKL		100	90
8	ACVRL1	TKL		92	93
9	AKT1	AGC		98	84
10	AKT2	AGC		94	87
11	AKT3	AGC		110	97
12	ALK	TK		92	87
13	AMPKalpha1	CAMK		108	103
14	ARAF YDYD	TKL		97	87
15	ARK5	CAMK		118	100
16	ASK1	STE		94	95
17	AuroraA	OTHER		96	101
18	AuroraB	OTHER		74	78
19	AuroraC	OTHER		92	84
20	AXL	TK		105	91
21	BLK	TK		95	90
22	BMPR1A	TKL		104	95
23	BMPR1B	TKL		94	88
24	BMX	TK		102	84
25	BRAF	TKL		95	81
26	BRK	TK		105	109
27	BRSK1	CAMK		88	84
28	BRSK2	CAMK		93	93
29	BTK	TK		99	90
30	BUB1B	OTHER		106	99
31	CAMK1D	CAMK		86	86
32	CAMK2A	CAMK		106	95
33	CAMK2B	CAMK		100	82
34	CAMK2D	CAMK		98	95
35	CAMK2G	CAMK		131	86
36	CAMK4	CAMK		90	88
37	CAMKK1	OTHER		94	96
38	CAMKK2	OTHER		99	97
39	CDC42BPA	AGC		120	89
40	CDC42BPB	AGC		94	84
41	CDC7/DBF4	OTHER		99	79
42	CDK1/CycA2	CMGC		97	88
43	CDK1/CycB1	CMGC		91	87
44	CDK1/CycE1	CMGC		106	96
45	CDK10/CycQ	CMGC		91	84
46	CDK11B/CycK	CMGC		93	89
47	CDK12/CycK	CMGC		83	84
48	CDK13/CycK	CMGC		108	96
49	CDK14/CycY	CMGC		105	100
50	CDK15/CycA2	CMGC		94	99
51	CDK15/CycB1	CMGC		99	99
52	CDK16/CycY	CMGC		102	97
53	CDK17/p35NCK	CMGC		98	81
54	CDK18/CycY	CMGC		93	87
55	CDK19/CycC	CMGC		90	79
56	CDK2/CycA2	CMGC		86	84
57	CDK2/CycD1	CMGC		96	105
58	CDK2/CycE1	CMGC		94	83
59	CDK20/CycH	CMGC		76	80
60	CDK20/CycT1	CMGC		93	81
61	CDK3/CycC	CMGC		100	93
62	CDK3/CycE1	CMGC		92	86
63	CDK4/CycD1	CMGC		87	88
64	CDK4/CycD2	CMGC		104	91
65	CDK4/CycD3	CMGC		118	98
66	CDK5/p25NCK	CMGC		99	94
67	CDK5/p35NCK	CMGC		84	92
68	CDK6/CycD1	CMGC		89	88
69	CDK6/CycD2	CMGC		97	94
70	CDK6/CycD3	CMGC		86	84
71	CDK7/CycH/MAT1	CMGC		109	88
72	CDK8/CycC	CMGC		84	86
73	CDK9/CycK	CMGC		91	85
74	CDK9/CycT1	CMGC		97	91
75	CHK1	CAMK		89	85
76	CHK2	CAMK		76	76
77	CK1alpha1	CK1		110	102
78	CK1delta	CK1		106	97
79	CK1epsilon	CK1		119	111
80	CK1gamma1	CK1		105	96
81	CK1gamma2	CK1		105	92
82	CK1gamma3	CK1		92	90
83	CK2alpha1	OTHER		26	14
84	CK2alpha2	OTHER		32	20
85	CLK1	CMGC		94	88
86	CLK2	CMGC		103	88
87	CLK3	CMGC		112	117
88	CLK4	CMGC		96	84
89	COT	STE		89	93
90	CSF1R	TK		104	93
91	CSK	TK		90	84
92	DAPK1	CAMK		21	11
93	DAPK2	CAMK		12	8
94	DAPK3	CAMK		21	7
95	DCAMKL2	CAMK		99	99
96	DDR2	TK		86	79
97	DMPK	AGC		101	95
98	DNAPK	ATYPICAL		100	92
99	DYRK1A	CMGC		82	72
100	DYRK1B	CMGC		85	78
101	DYRK2	CMGC		79	73
102	DYRK3	CMGC		85	76
103	DYRK4	CMGC		94	95
104	EEF2K	ATYPICAL		90	94
105	EGFR	TK		82	66
106	EIF2AK2	OTHER		81	75
107	EIF2AK3	OTHER		87	96
108	EIF2AK4	OTHER		93	80
109	EPHA1	TK		96	87
110	EPHA2	TK		97	74
111	EPHA3	TK		109	92
112	EPHA4	TK		102	101
113	EPHA5	TK		95	84
114	EPHA6	TK		98	87
115	EPHA7	TK		97	89
116	EPHA8	TK		82	104
117	EPHB1	TK		100	85
118	EPHB2	TK		80	79
119	EPHB3	TK		109	103
120	EPHB4	TK		109	103
121	ERBB2	TK		103	88
122	ERBB4	TK		96	92
123	ERK1	CMGC		85	80
124	ERK2	CMGC		85	77
125	ERK5	CMGC		89	75
126	ERK7	CMGC		92	90
127	FAK	TK		121	97
128	FER	TK		112	88
129	FES	TK		95	87
130	FGFR1	TK		95	98
131	FGFR2	TK		93	89
132	FGFR3	TK		101	83
133	FGFR4	TK		92	86
134	FGR	TK		89	91
135	FLT3	TK		88	85
136	FRK	TK		102	94
137	FYN	TK		119	105
138	GRK2	AGC		103	105
139	GRK3	AGC		106	104
140	GRK4	AGC		114	102
141	GRK5	AGC		113	102
142	GRK6	AGC		89	88
143	GRK7	AGC		113	105
144	GSG2	OTHER		93	91
145	GSK3alpha	CMGC		92	86
146	GSK3beta	CMGC		96	86
147	HCK	TK		91	79
148	HIPK1	CMGC		99	99
149	HIPK2	CMGC		96	86
150	HIPK3	CMGC		115	96
151	HIPK4	CMGC		91	87
152	HRI	OTHER		102	102
153	IGF1R	TK		95	92
154	IKKalpha	OTHER		85	79
155	IKKbeta	OTHER		97	82
156	IKKepsilon	OTHER		84	90
157	INSR	TK		94	93
158	INSRR	TK		92	89
159	IRAK1	TKL		99	85
160	IRAK4	TKL		87	86
161	ITK	TK		98	97
162	JAK1	TK		104	89
163	JAK2	TK		101	102
164	JAK3	TK		91	89
165	JNK1	CMGC		96	89
166	JNK2	CMGC		93	90
167	JNK3	CMGC		98	88
168	KIT	TK		99	90
169	LCK	TK		93	85
170	LIMK1	TKL		104	94
171	LIMK2	TKL		92	88
172	LKB1/MO25a/STRADa	CAMK		112	98
173	LRRK2	TKL		94	85
174	LTK	TK		99	93
175	LYN	TK		96	84
176	MAP3K1	STE		90	88
177	MAP3K10	STE		95	89
178	MAP3K11	STE		93	82
179	MAP3K7/MAP3K7IP1	STE		96	99
180	MAP3K9	STE		109	100
181	MAP4K2	STE		91	88
182	MAP4K4	STE		97	88
183	MAP4K5	STE		103	106
184	MAPKAPK2	CAMK		121	93
185	MAPKAPK3	CAMK		106	88
186	MAPKAPK5	CAMK		95	93
187	MARK1	CAMK		93	93
188	MARK2	CAMK		109	100
189	MARK3	CAMK		98	94
190	MARK4	CAMK		108	104
191	MASTL	AGC		89	79
192	MATK	TK		98	93
193	MEK1	STE		96	85
194	MEK2	STE		96	80
195	MEK5	STE		94	80
196	MEKK2	STE		104	98
197	MEKK3	STE		103	97
198	MELK	CAMK		85	79
199	MERTK	TK		88	87
200	MET	TK		95	91
201	MINK1	STE		90	83
202	MKK4	STE		86	101
203	MKK6 SDTD	STE		82	83
204	MKK7	STE		104	102
205	MKNK1	CAMK		100	95
206	MKNK2	CAMK		99	90
207	MLK4	TKL		115	99
208	MST1	STE		104	90
209	MST2	STE		98	82
210	MST3	STE		117	106
211	MST4	STE		97	84
212	MTOR	ATYPICAL		95	105
213	MUSK	TK		93	93
214	MYLK	CAMK		74	78
215	MYLK2	CAMK		83	77
216	MYLK3	CAMK		88	93
217	NDR1	AGC		96	96
218	NDR2	AGC		101	94
219	NEK1	OTHER		90	80
220	NEK11	OTHER		94	89
221	NEK2	OTHER		102	93
222	NEK3	OTHER		108	101
223	NEK4	OTHER		90	91
224	NEK6	OTHER		94	92
225	NEK7	OTHER		104	100
226	NEK9	OTHER		94	92
227	NIK	STE		95	91
228	NLK	CMGC		95	94
229	p38alpha	CMGC		85	67
230	p38beta	CMGC		78	70
231	p38delta	CMGC		99	93
232	p38gamma	CMGC		105	92
233	PAK1	STE		101	93
234	PAK2	STE		93	95
235	PAK3	STE		94	89
236	PAK4	STE		77	86
237	PAK6	STE		95	93
238	PAK7	STE		85	76
239	PASK	CAMK		78	72
240	PBK	OTHER		104	104
241	PDGFRalpha	TK		102	96
242	PDGFRbeta	TK		90	85
243	PDK1	AGC		100	90
244	PHKG1	CAMK		94	91
245	PHKG2	CAMK		102	91
246	PIM1	CAMK		25	17
247	PIM2	CAMK		97	73
248	PIM3	CAMK		30	19
249	PKA	AGC		104	88
250	PKCalpha	AGC		99	90
251	PKCbeta1	AGC		94	87
252	PKCbeta2	AGC		85	87
253	PKCdelta	AGC		103	91
254	PKCepsilon	AGC		103	110
255	PKCeta	AGC		118	97
256	PKCgamma	AGC		100	91
257	PKCiota	AGC		87	120
258	PKCmu	AGC		88	83
259	PKCnu	AGC		96	90
260	PKCtheta	AGC		89	91
261	PKCzeta	AGC		100	92
262	PKMzeta	AGC		104	83
263	PKN3	AGC		96	96
264	PLK1	OTHER		98	94
265	PLK3	OTHER		94	100
266	PRK1	AGC		100	92
267	PRK2	AGC		82	83
268	PRKD2	CAMK		87	78
269	PRKG1	AGC		92	89
270	PRKG2	AGC		97	94
271	PRKX	AGC		102	79
272	PYK2	TK		94	90
273	RAF1 YDYD	TKL		89	80
274	RET	TK		94	83
275	RIPK2	TKL		95	106
276	RIPK4	TKL		87	94
277	RIPK5	TKL		95	92
278	ROCK1	AGC		94	89
279	ROCK2	AGC		89	88
280	RON	TK		101	94
281	ROS	TK		96	94
282	RPS6KA1	AGC		97	79
283	RPS6KA2	AGC		86	80
284	RPS6KA3	AGC		91	74
285	RPS6KA4	AGC		103	88
286	RPS6KA5	AGC		98	95
287	RPS6KA6	AGC		99	95
288	S6K	AGC		92	96
289	S6Kbeta	AGC		88	88
290	SAK	OTHER		81	75
291	SGK1	AGC		105	87
292	SGK2	AGC		102	94
293	SGK3	AGC		96	99
294	SIK1	CAMK		82	85
295	SIK2	CAMK		95	86
296	SIK3	CAMK		86	82
297	SLK	STE		84	86
298	SNARK	CAMK		98	97
299	SNK	OTHER		95	105
300	SRC	TK		104	89
301	SRMS	TK		102	113
302	SRPK1	CMGC		96	87
303	SRPK2	CMGC		98	99
304	STK17A	CAMK		89	63
305	STK17B	CAMK		50	46
306	STK23	CAMK		91	87
307	STK25	STE		98	92
308	STK33	CAMK		97	111
309	STK39	STE		92	86
310	SYK	TK		94	89
311	TAOK2	STE		101	94
312	TAOK3	STE		91	94
313	TBK1	OTHER		102	92
314	TEC	TK		95	96
315	TGFBR1	TKL		94	89
316	TGFBR2	TKL		81	75
317	TIE2	TK		111	95
318	TLK1	AGC		117	94
319	TLK2	AGC		116	106
320	TNK1	TK		101	90
321	TRKA	TK		104	89
322	TRKB	TK		106	97
323	TRKC	TK		100	74
324	TSF1	OTHER		91	94
325	TSK2	CAMK		107	100
326	TSSK1	CAMK		87	77
327	TTBK1	CK1		97	88
328	TTBK2	CK1		96	94
329	TTK	OTHER		90	81
330	TXK	TK		86	85
331	TYK2	TK		95	87
332	TYRO3	TK		91	92
333	ULK2	OTHER		95	92
334	VEGFR1	TK		96	96
335	VEGFR2	TK		92	89
336	VEGFR3	TK		104	99
337	VRK1	CK1		102	91
338	VRK2	CK1		103	98
339	WEE1	OTHER		95	100
340	WNK1	OTHER		86	90
341	WNK2	OTHER		94	92
342	WNK3	OTHER		99	97
343	YES	TK		96	83
344	ZAK	TKL		96	96
345	ZAP70	TK		114	100
**Selectivity Score (< 50 % residual activity):**				0,020	0,023

***Classification of protein kinase families (Manning et al. Science 6 December 2002: Vol. 298 no. 5600 pp. 1912-1934):**
AGC: containing PKA, PKG and PKC families
CAMK: Calcium/Calmoduline-dependent protein kinases
CK1: Casein kinase 1 -like
CMGC: containing CDK, MAPK ,GSK3 and CLK families
TK: Tyrosine Kinase
TKL: Tyrosine Kinase-like
STE: Homologs of Yeast Sterile 7, Sterile 11, Sterile 20 Kinases

MKK6 SDTD, RAF1 YDYD, ARAF YDYD: Constitutively active variants

Supplementary Table 9. Top ten drugs with highest Spearman rho coefficient correlations and lowest Benjamini & Hochberg adjusted p-values.

Rank	Compound	Mode Of Action	ρ (Spearman test)	p-value	BH adjusted p-value	Cell line (n=)
1	Docetaxel	Inhibitor of microtubule assembly, Mitosis	0.67	2.69E-14	1.23E-11	98
2	Vincristine	Inhibitor of microtubule assembly, Mitosis	0.67	7.11E-15	6.52E-12	104
3	Podophyllotoxin bromide	Inhibitor of microtubule assembly	0.65	6.33E-14	1.93E-11	104
4	Vinblastine	Inhibitor of microtubule assembly, Mitosis	0.61	1.12E-12	2.06E-10	110
5	SB225002	Inhibitor of chemokine receptor 2	0.57	8.08E-13	1.85E-10	135
6	Eg5_9814	KIF11 inhibitor	0.56	4.08E-10	4.16E-08	105
7	Telomerase Inhibitor IX	Telomerase inhibitor	0.56	2.45E-10	3.75E-08	110
8	YK-4-279	Inhibitor of RNA helicase A (RHA) binding to EWS-FLI1, inhibitor of ERG and ETV1 activity	0.53	2.96E-09	1.81E-07	110
9	Parbendazole	Inhibitor of microtubule assembly	0.52	2.48E-10	3.25E-08	131
10	GSK461364	PLK1 inhibitor, Mitosis, Cell Cycle	0.52	2.77E-10	3.18E-08	131

Supplementary Table 10. CK2-TN03 permeability in MDCKII-MDR1 cells

	- P-gp inhibitor (elacridar)			+ P-gp inhibitor (elacridar)		
	**A2B**	**B2A**	**Efflux ratio**	**A2B**	**B2A**	**Efflux ratio**
**Compound ID**	P_{app} (10^{-6} cm/sec)	P_{app} (10^{-6} cm/sec)		P_{app} (10^{-6} cm/sec)	P_{app} (10^{-6} cm/sec)	
Amprenavir	0.7	20.6	29.5	6.4	18.3	2.9
Diclofenac	12.4	28.5	2.3	14.4	24.1	1.7
**CK2-TN03**	6.6	0.7	0.1	8.4	0.8	0.1

Amprenavir used as control for low permeability and as P-gp substrate. Diclofenac used as control for high permeability and as not a P-gp substrate.

Supplementary Table 11. Clearance in mouse liver microsomes.

**Compound ID**	**%remaining at 1h**	**Half-life (min)**	**Clint [µl/min/mg]**	**Pred in vivo hep CL [ml/min/kg]**	**Pred in vivo hep CL [%LBF]**	**Clearance classification**
**CK2-TN03**	8.9	8.8	158.6	111.0	85	HIGH
Testosterone	0.0	9.2	150.3	110.1	84	HIGH
Propranolol	9.6	8.1	170.5	112.2	86	HIGH
Caffeine	103.8	>60	<12.5	<39.9	<30	LOW

Testosterone and propranolol used as controls for high clearance and caffeine used as control for low clearance.

Supplementary Table 12. Protein binding in mouse plasma.

**Compound ID**	**Fb (%)_A**	**Fb (%)_B**	**Fb (%) AVG**	**Classification**
**CK2-TN03**	99.2	99.1	**99.2**	HIGH binding
Nicardapine	99.5	99.6	99.5	HIGH binding
Verapamil	85.8	87.6	86.7	MODERATE binding
Caffeine	21.5	22.3	21.9	LOW binding

Niardapine, verapamil and caffeine used as controls for high, moderate and low binding, respectively.

Supplementary Table 13. Pharmacokinetic profiling of CK2-T03 following intravenous administration.

**Animal ID**	**Dose (mg/kg)**	**#t½ (hr)**	***t½ (hr)**	**C_0 (ng/mL)**	**AUC (hr*ng/mL)**	**CL mL/min/kg**	**V_{ss} L/kg**
**1**	10	0.146	5.22	9437	887	188	16.5
**2**	10	0.275	5.49	10364	948	176	14.3
**3**	10	0.157	4.37	6724	653	255	33.5
**Mean**		**0.193**	**5.03**	**8842**	**829**	**206**	**21.4**
**SD**		**0.072**	**0.584**	**1891**	**156**	**42.8**	**10.5**
**CV**		**37**	**12**	**21**	**19**	**21**	**49**

#initial phase *terminal phase

Supplementary Table 14. Tested CK2-T03 analogues.

Compound	Structure	Compound	Structure
**TN11**	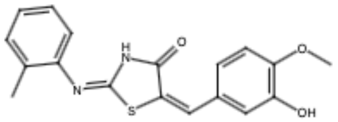	**TN12**	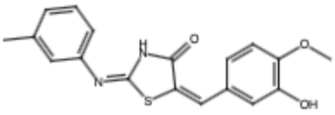
**TN13**	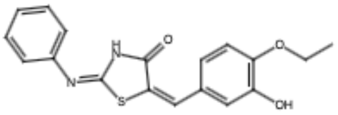	**TN14**	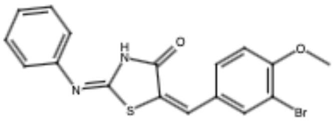
**TN15**	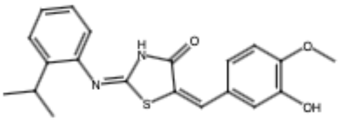	**TN16**	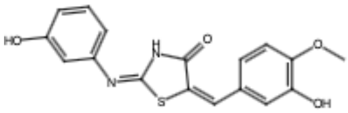
**TN17**	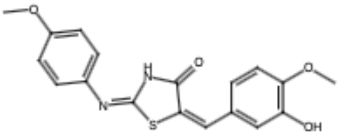	**TN18**	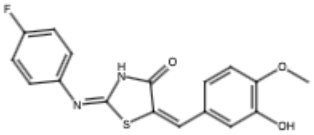
**TN19**	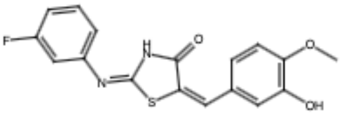	**TN20**	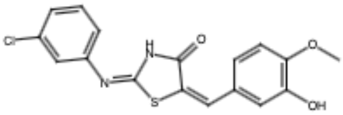
**TN21**	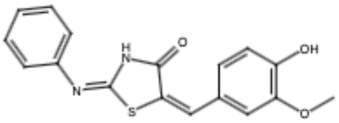	**TN22**	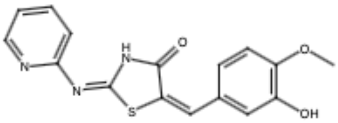
**TN23**	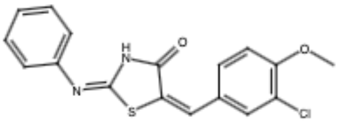	**TN24**	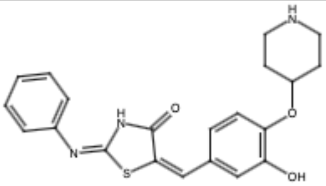
**TN25**	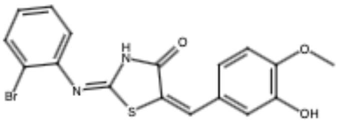		

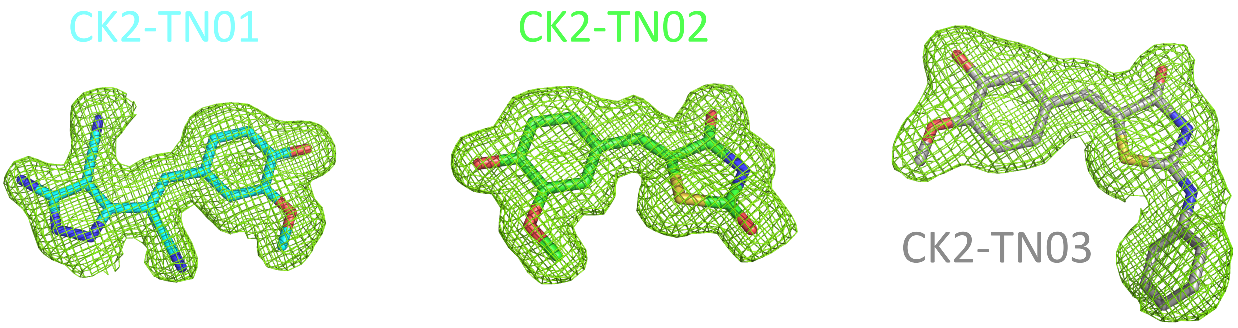

Supplementary Figure 1. **Electron densities for the reported compounds.** F_o-F_c polder OMIT maps (green) are contoured at 3.0 σ (Liebschner *et al.* 2017).

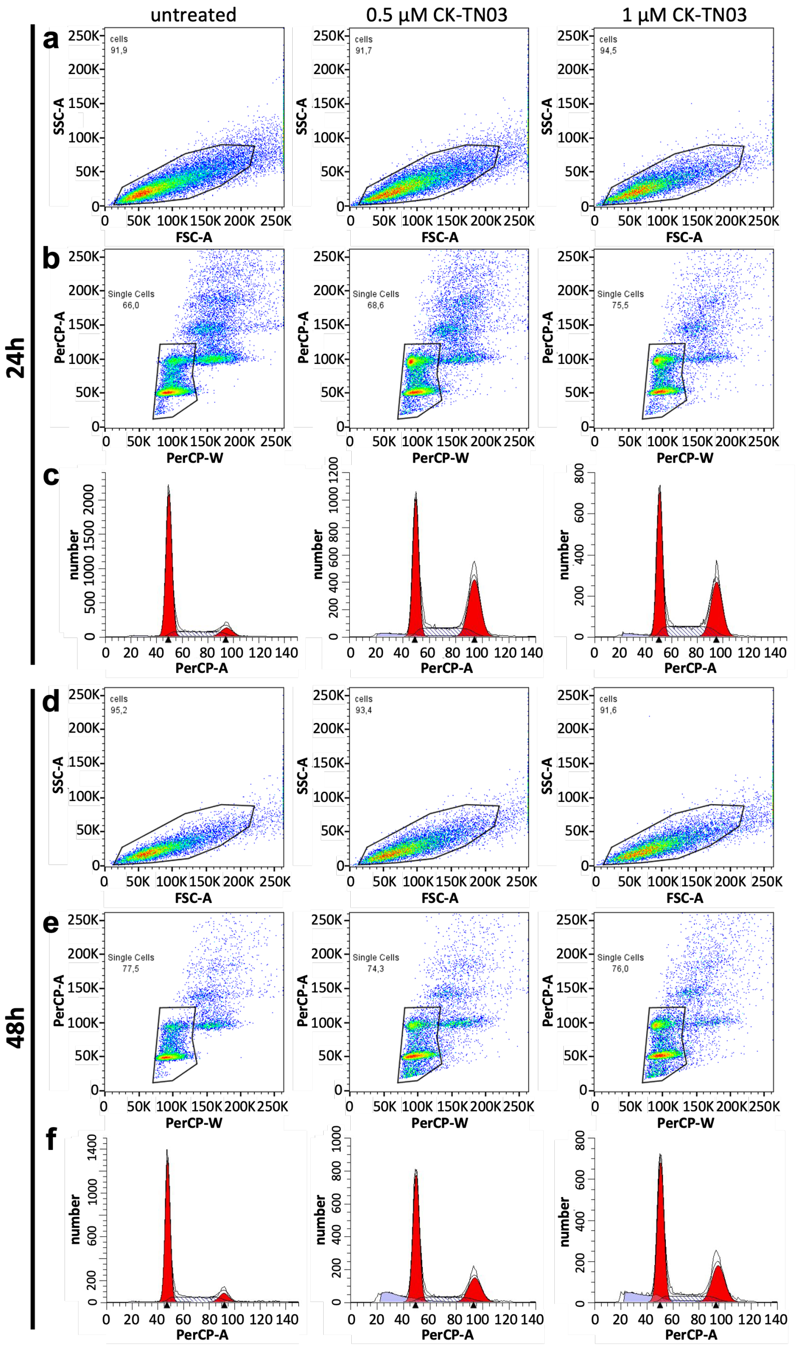

Supplementary Figure 2. Contour plot and linear PI plot of cell cycle analysis of CHP-212. The image shows three plots (representative images from one of the four independent replicates) of the cell cycle analysis of CHP-212 treated for 24h (**a, b** and **c**) or 48h (**d**, **e** and **f**) with 0.5 µM (second column) and 1 µM CK2-TN03 (third column). The first rows for each time point (A and D) show the FSC-A vs. SSC-A plots, the second rows (B and E) the PerCP-W vs. PerCP-A and the last rows (C and F) the PI intensity (PerCP-A) vs. the number of events. Plots in rows **a**, **b**, **d** and **e** were obtained using the FlowJo™ software, while rows **c** and **f** plots using ModFit™ software.

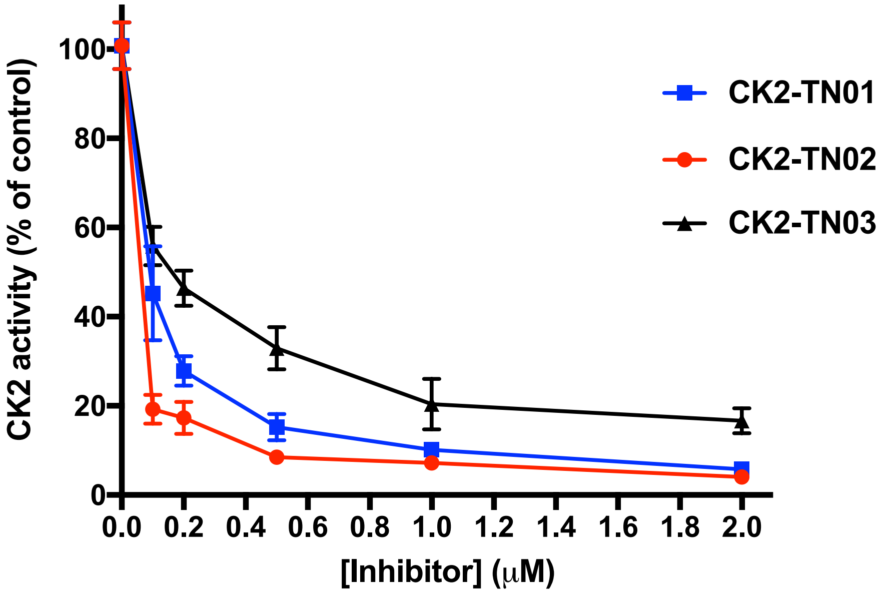

Supplementary Figure 3. Effect of CK2-TN01, CK2-TN02 and CK2-TN03 on CK2 activity. Recombinant CK2α activity was measured towards the model peptide CK2-tide, in the presence of increasing concentrations of the inhibitors, or of the vehicle (DMSO) for the controls. Activity is reported in percentage, assigning 100 % to the control. The means ± SEM of 4 to 8 independent experiments are shown. The curves were analysed by the GraphPad Prism 7.0a software for the calculation of the IC50.

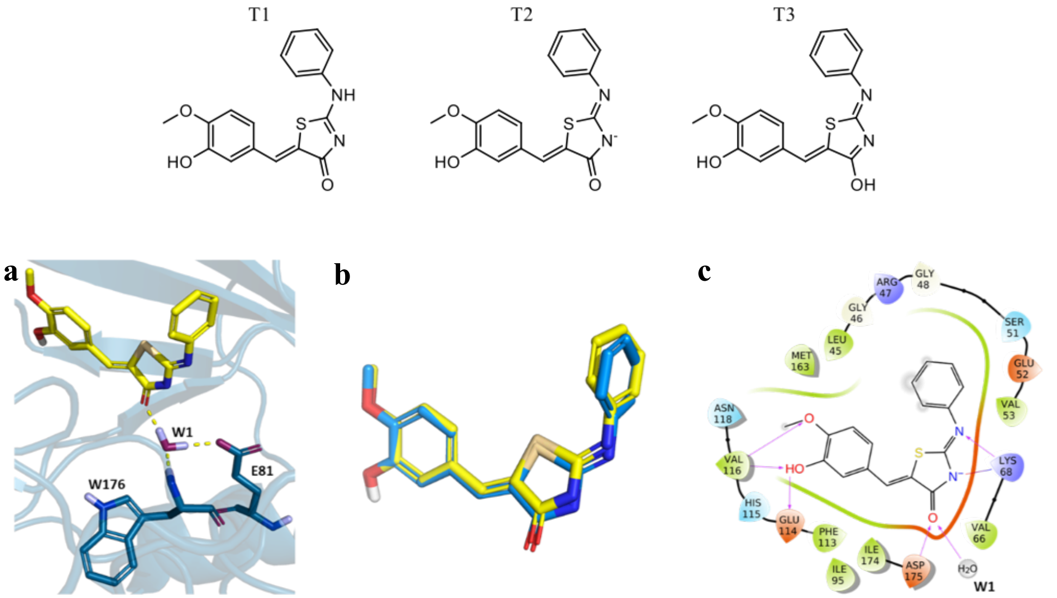

Supplementary Figure 4. CK2-TN03 tautomerism. The three analysed tautomers are shown in the top panel. a) Water mediated interactions established between TN03-T2, Glu81 and Trp176. b) Superimposition between the experimental and docked pose of TN03-T2 within CK2 protein. c) Simplified 2D representation of the predicted interaction between TN03-T2 and CK2 residues.

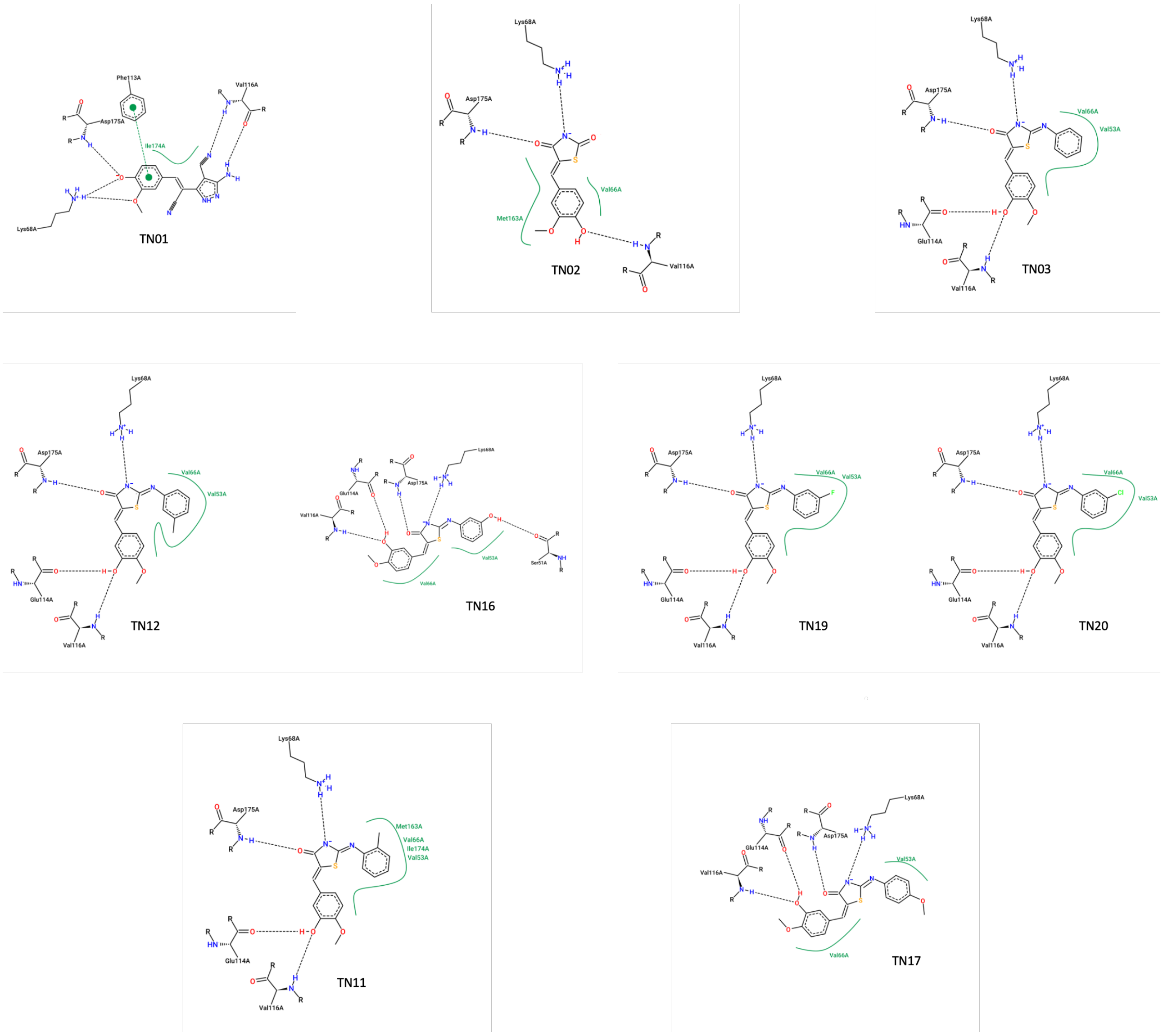

Supplementary Figure 5. Detailed interactions of the TN compound series in the CK2 pocket. Diagrams were generated with PoseView (Stierand *et al.,* 2010).

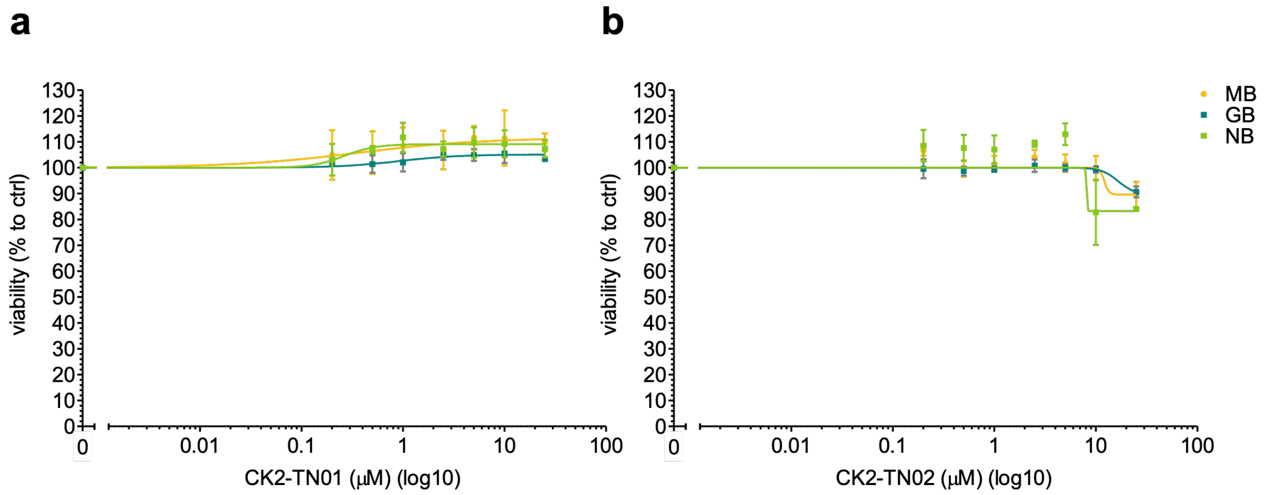

Supplementary Figure 6. Effect on cellular viability of CK2-TN01 and CK2-TN02. Cell viability of MB cell line DAOY (yellow), GB cell line U87 (blue) and NB cell line CHP-212 (green), measured by Alamar blue assay, after 48h of exposure to increasing concentrations (0.2 to 25 μM) of CK2-TN01 (**a**) and CK2-TN02 (**b**). Each point represents the mean ± SD of 3 independent experiments.

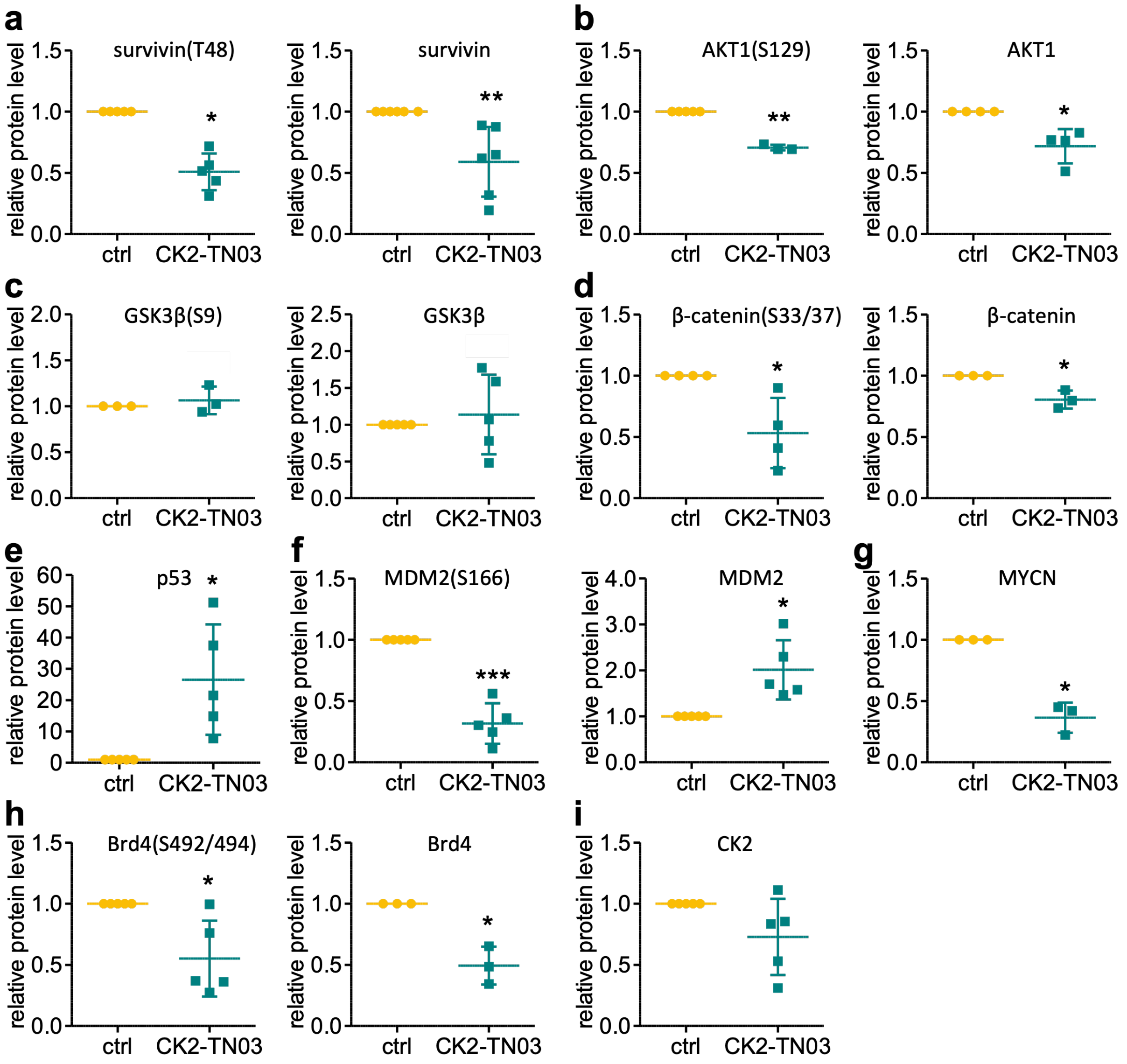

Supplementary Figure 7. Quantification of western blot experiments. The graphs represent the relative intensity (compared to the untreated sample and normalized to a-actinin) of the bands obtained in the western blot experiments shown in Fig. 4 for the following proteins: survivin and phospho-survivin (T48) (a); AKT1 and phospho-AKT1(S129) (b); GSK3β and phospho-GSK3β(S9) (c); β-catenin and phospho-β-catenin(S33/37) (d); p53 (e); MDM2 and phospho-MDM2(S166) (f); MYCN (g); Brd4 and phospho-Brd4(S492/494) (h); CK2 (i). The graphs show the mean ± SD of 3 to 6 independent experiments. ns = $P > 0.05$ (phospho-GSK3β(S9) P=0.5356; GSK3β P=0.5984; CK2 P=0.1227), *$P < 0.05$ (survivin P=0.0169; AKT1 P=0.027; phospho-β-catenin(S33/37) P=0.0473; β-catenin P=0.0447; p53 P=0.0315; MDM2 P=0.0249; MYNC P=0.0123; phospho-Brd4(S492/494) P=0.0321; Brd4 P=0.0296), **$P < 0.01$ (phospho-survivin(T8) P=0.0019; phospo-AKT1(S129) P=0.0021), ***$P < 0.001$ (phospho-MDM2(S166) P=0.0008), two-tailed t-test.

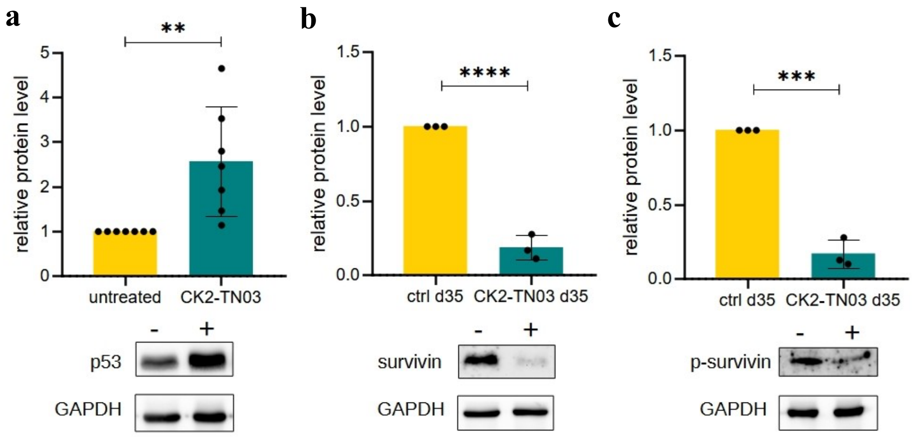

Supplementary Figure 8. p53 and survivin protein level in the engrafted tumors. Western blot analysis of the total protein level of p53 (a) and survivin (b) and of the phosphorylation level of survivin (T48) (c). A representative image is shown out of 3 to 6 technical experiments. The proteins were extracted from one untreated animal (-) or an animal treated with CK2-TN03 (+). The tumors were collected 35 days after the beginning of treatment (lasting 28 days). The graphs show the mean ± SD of 3 to 6 technical replicates. **P < 0.01, ***P < 0.001, unpaired t-test, ****P < 0.0001, unpaired t-test.

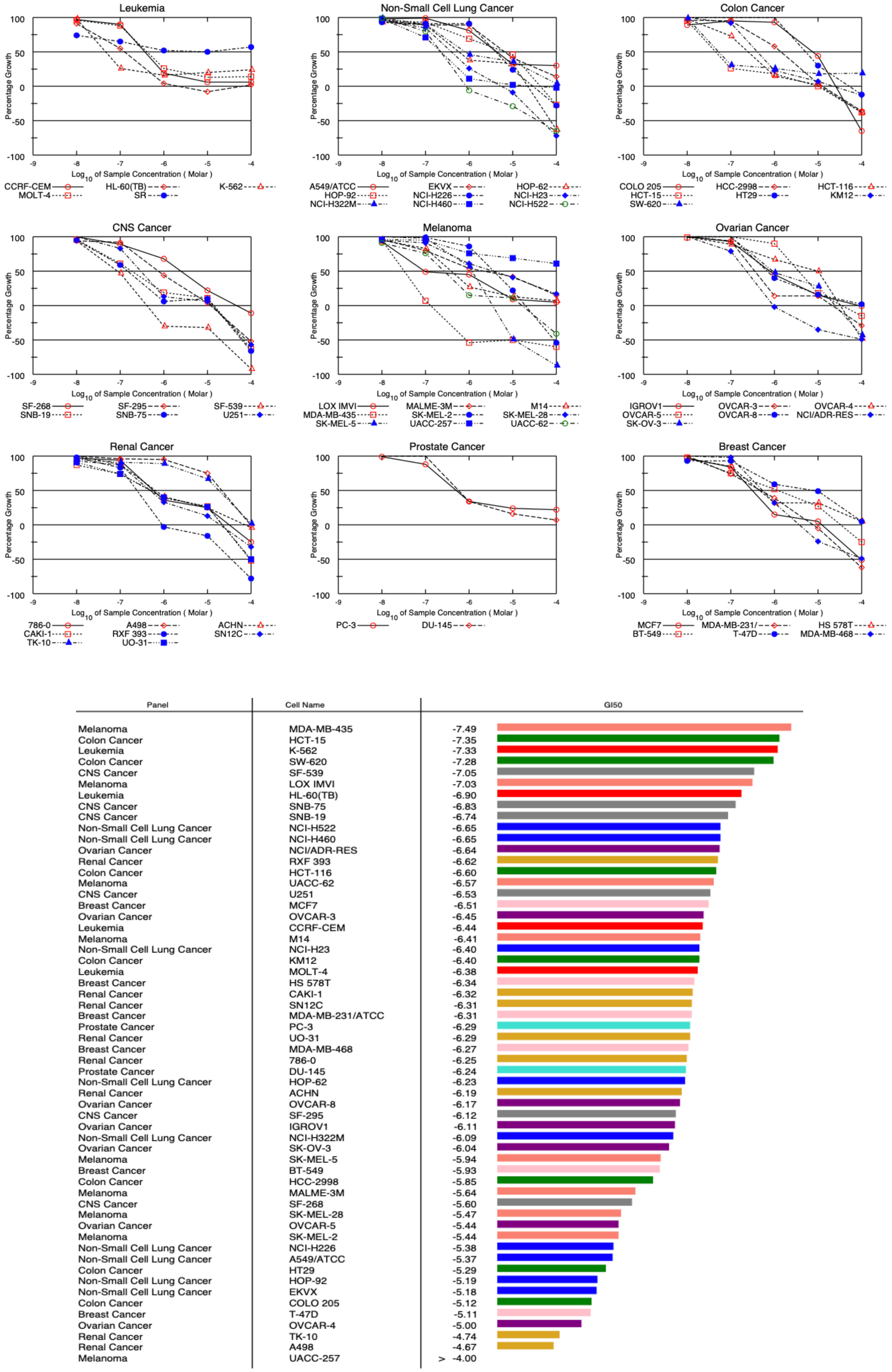

Supplementary Figure 9. **Growth curves and log GI$_{50}$ waterfall graph for CK2-TN03 activity on the NCI-60 panel.**

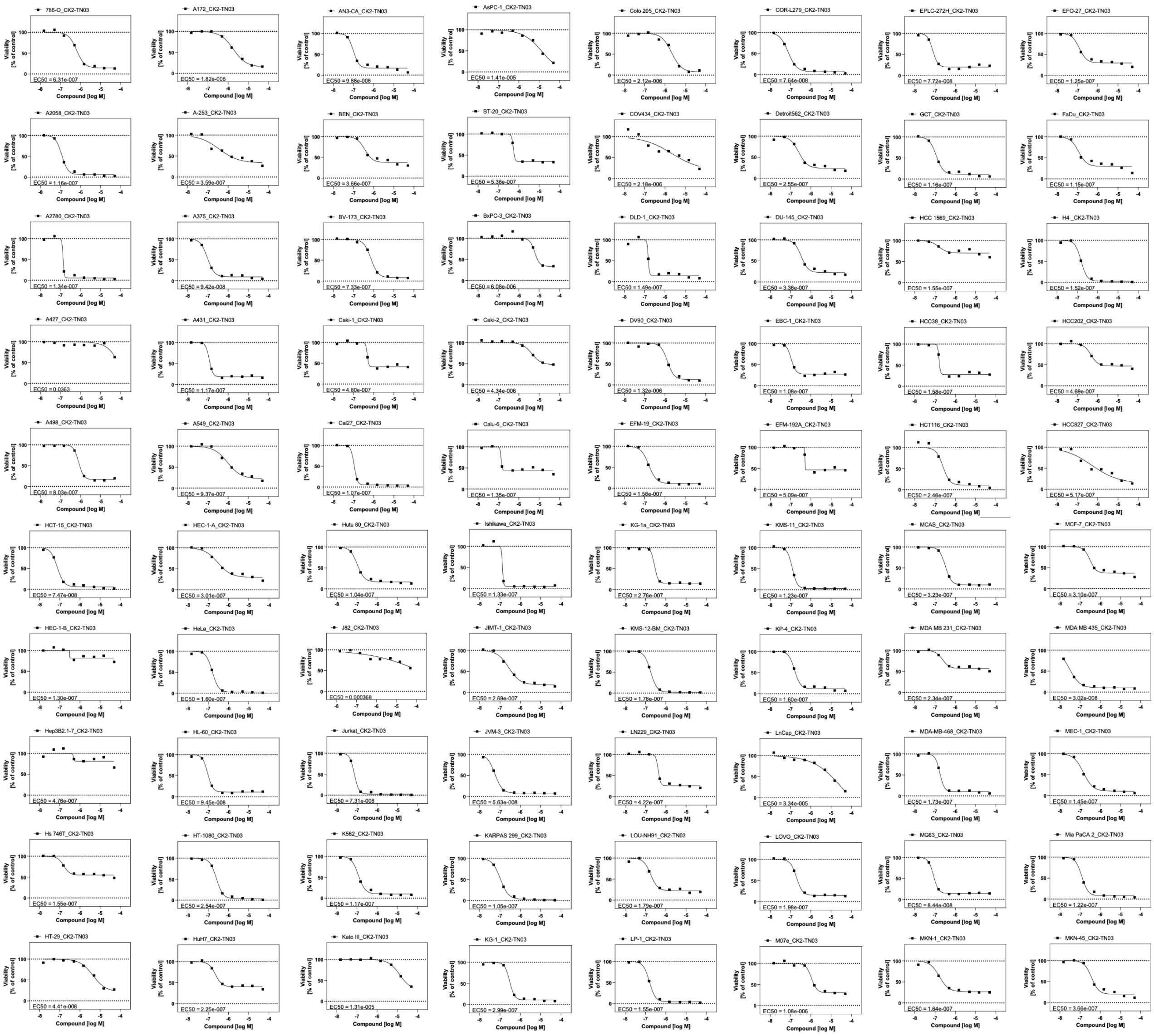

Supplementary Figure 10. **Effect on cellular viability of CK2-TN03.** Cell lines 1-80 from the ProLiFiler panel are shown.

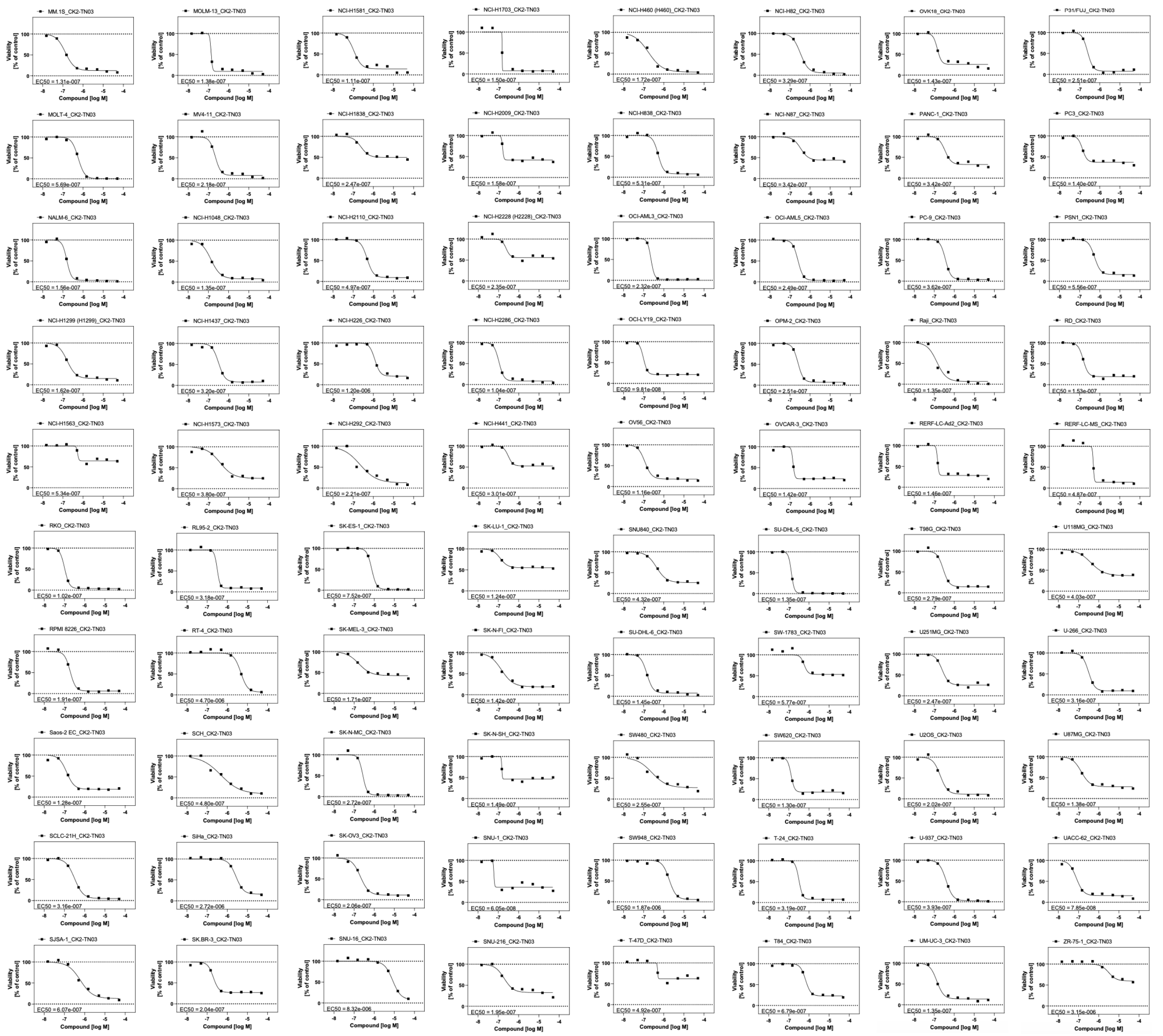

Supplementary Figure 11. **Effect on cellular viability of CK2-TN03.** Cell lines 81-160 from the ProLiFiler panel are shown.

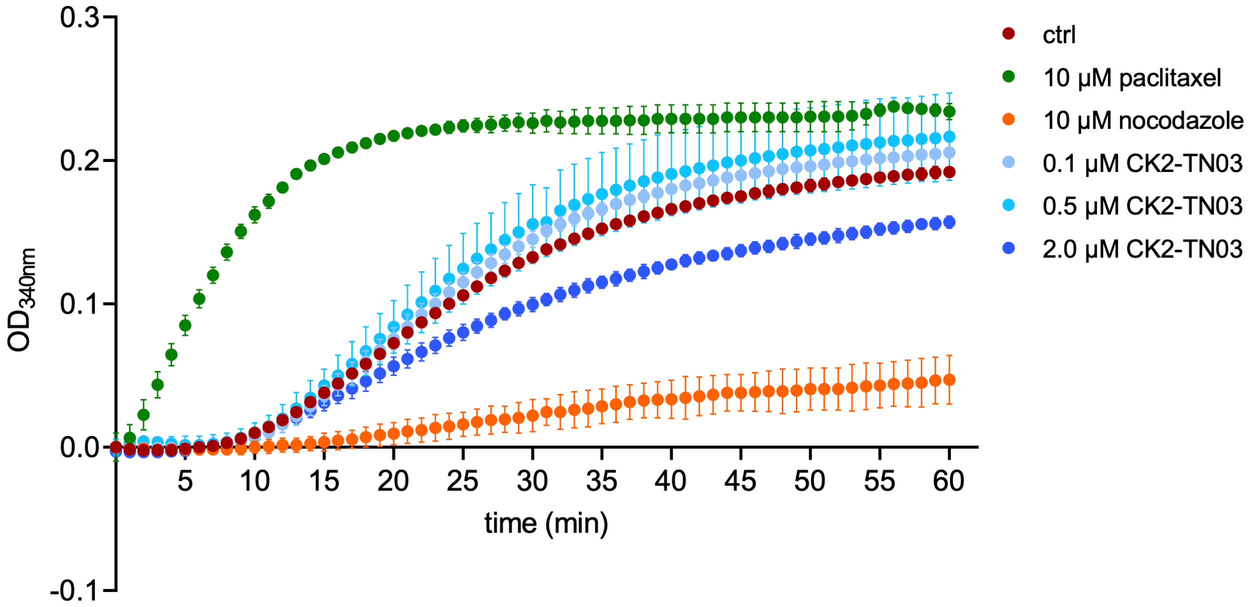

Supplementary Figure 12. **Tubulin polymerization assay.** The assay has been performed following manufacturer instructions (Cytoskeleton Inc., tubulin polymerization kit cat. BK006P). CK2-TN03 has been tested at 100 nM (median EC_{50} for the most affected cancer entity, melanoma), 500 nM (above effective concentration for most cell lines, median EC_{50} for all cancer entities = 295 nM) and 2 µM.

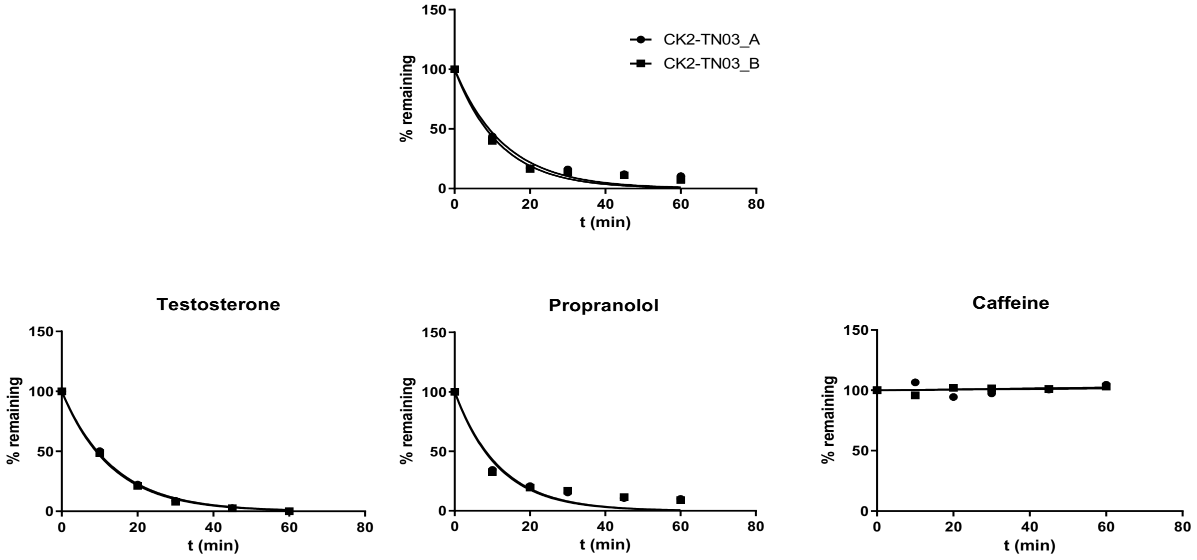

Supplementary Figure 13. **CK2_TN03 clearance in liver microsomes.** Incubation has been conducted at 37°C with 1 µM initial compound concentration.

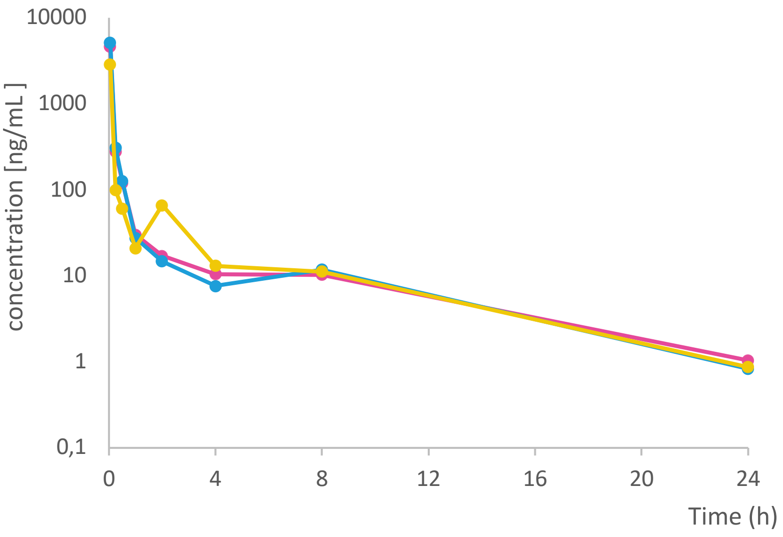

Supplementary Figure 14. **Pharmacokinetic profiling of CK2-TN03 following intravenous administration.** Each curve refer to a different animal.

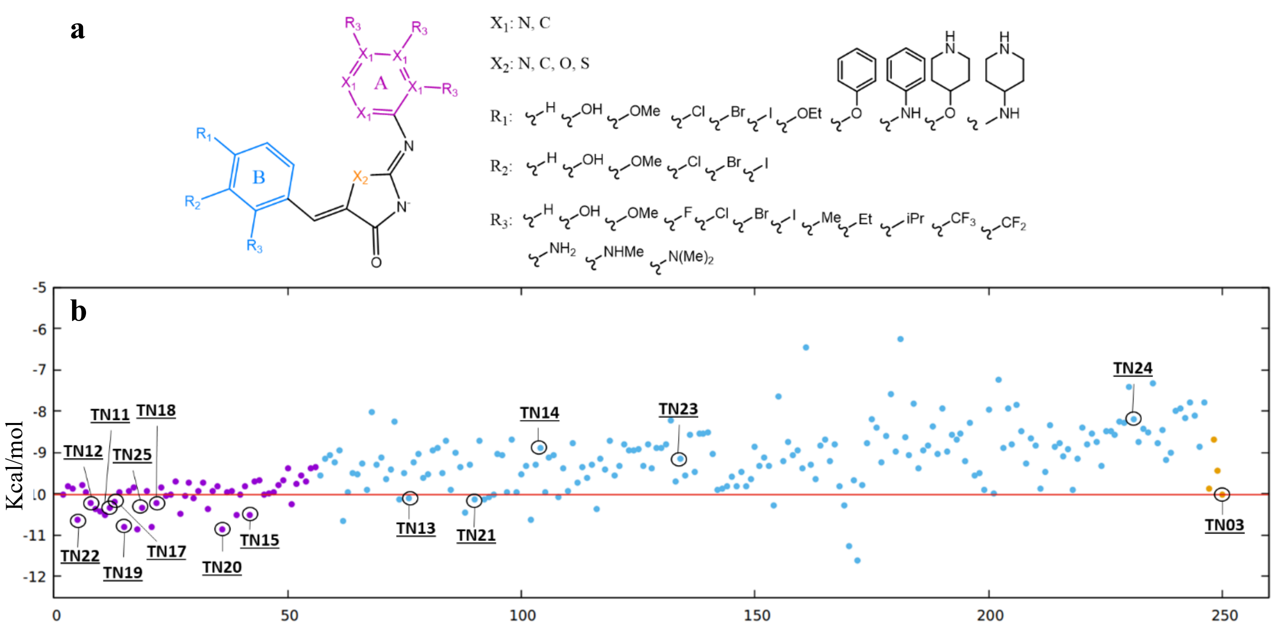

Supplementary Figure 15. **Generation and investigation of CK2-TN03 analogues as CK2 binders.** a) Starting from the parent compound CK2-TN03, two virtual focused libraries exploring the 4-thiazolidinone substituents (i.e. rings A and B) were created. Specifically, single modification on each ring allowed the generation of two sets composed by 54 (Ring A set) and 190 (Ring B set) compounds. Additionally, the effect of the replacement of the ring sulfur atom with N, C, and O (core analogues, 3 compounds) was investigated. b) The 247 generated compounds were submitted to molecular docking experiments. Computed docking score for the derivatives composing the focused libraries (magenta and blue dots for Ring A and B sets, respectively) and for core analogues (orange dots) are shown.

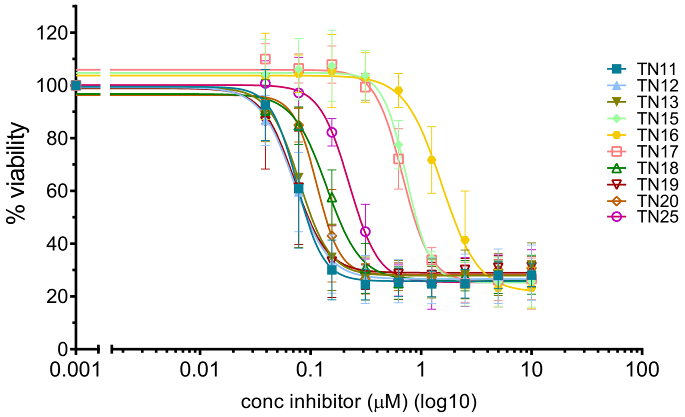

Supplementary Figure 16. **Effect of CK2-TN03 analogues on the viability of CHP-212 NB cell line.** Comparison between the CHP-212 cell line viability caused by the treatment with 11 different CK2 kinase inhibitors, measured by Alamar blue assay, after 48h of exposure to increasing concentrations of the inhibitors (0.039 to 10 µM). Each point represents the mean ± SD of 4 independent experiments.

REFERENCES

- Harenza J.L. et al. Transcriptomic profiling of 39 commonly-used neuroblastoma cell lines. *Sci. Data* 4, 170033 (2017).

- Lastowska M. et al. Comprehensive genetic and histopathologic study reveals three types of neuroblastoma tumors. *J. Clin. Oncol.* 19, 3080-3090 (2001).

- Liebschner D. et al. Polder maps: improving OMIT maps by excluding bulk solvent. *Acta Crystallogr. D Struct. Biol.* 73, 148-157 (2017).

- Stierand K. & Rarey, M. Drawing the PDB: Protein-Ligand Complexes in Two Dimensions. *ACS Med. Chem. Lett.* 1, 540-545 (2010).
